# A comparative study on DeepSeek and ChatGPT for bone and soft tissue tumor clinical practice

**DOI:** 10.3389/fonc.2025.1642880

**Published:** 2026-01-09

**Authors:** Baicheng Yang, Zhangfu Li, Xinxin Zhang, Xiaoyang Li, Ge Zhao, Yongli Jia, Shengji Yu

**Affiliations:** 1 Department of Orthopedics, Cancer Hospital Chinese Academy of Medical Sciences, Beijing, China; 2 The First Affiliated Hospital of Hebei North University, Zhangjiakou, China

**Keywords:** bone and soft tissue tumors, clinical practice, artificial intelligence, diagnostic accuracy, large language models

## Abstract

**Background:**

Artificial intelligence (AI) models are increasingly applied in clinical oncology, yet their comparative utility in specialized domains like bone and soft tissue tumors remains understudied. This study evaluates the diagnostic accuracy and clinical reasoning capabilities of DeepSeek and ChatGPT.

**Methods:**

A two-phase evaluation framework was implemented. First, 249 validated clinical questions (191 single-choice, 58 multiple-choice) spanning five domains (diagnosis, imaging, pathology, staging, treatment) were administered, with expert-derived answers serving as ground truth. Second, nine blinded clinicians scored model-generated analyses of a complex sarcoma case across seven clinical dimensions. Statistical analysis employed chi-square tests for accuracy comparisons, Cohen’s kappa for inter-rater reliability, and independent t-tests for expert ratings (α = 0.05).

**Results:**

DeepSeek outperformed ChatGPT in overall accuracy (74.7% vs 55.4%, p < 0.001), excelling in single-choice questions (86.9% vs 64.9%, p < 0.001) and two key domains: Pathology & Genetics (72.5% vs 40.0%, p = 0.006) and Treatment (71.3% vs 51.2%, p = 0.015). Experts rated DeepSeek higher in imaging interpretation (7.11 vs. 6.00, p = 0.002) and overall case analysis (54.11 vs. 51.56, p = 0.022). Cross-model analysis revealed DeepSeek uniquely answered 60 questions correctly where ChatGPT erred, while both models shared 51 errors.

**Conclusions:**

DeepSeek outperforms ChatGPT in diagnostic accuracy and specialized clinical reasoning for bone/soft tissue tumors, particularly in pathology and treatment domains. The significant performance gap (p < 0.001) and 24.1% unique correct responses position DeepSeek as a more reliable diagnostic aid, though shared errors (51 questions) necessitate hybrid AI-clinician workflows.

## 1 Introduction

Integrating artificial intelligence (AI) in clinical oncology has revolutionized diagnostic accuracy, treatment planning, and patient management (1, 2). Large language models (LLMs) like OpenAI’s ChatGPT and DeepSeek-AI’s DeepSeek-R1 have emerged as transformative tools, offering rapid data analysis and decision support in complex medical scenarios (3, 4). Recent studies highlight their potential to address challenges such as diagnostic variability and limited access to specialized care, particularly in resource-constrained settings (5, 6). For instance, ChatGPT’s integration into ophthalmic workflows has demonstrated its ability to streamline electronic medical record documentation, including automated summarization of imaging findings, laboratory data, and clinical histories, enhancing efficiency in multidisciplinary eye care (7). Similarly, DeepSeek’s open-source framework has demonstrated potential in clinical decision support and patient education through AI-driven case simulations. However, its diagnostic reliability in high-stakes oncology scenarios requires rigorous validation to address risks of hallucinations and data privacy compliance (8). These advancements underscore the growing role of AI in augmenting clinical workflows, yet comparative evaluations of LLMs in specialized domains like bone and soft tissue tumors are scarce, necessitating rigorous benchmarking to guide practical implementation.

Bone and soft tissue tumors epitomize the diagnostic and therapeutic challenges of rare malignancies. Characterized by histopathological mimicry, molecular heterogeneity (e.g., over 100 sarcoma subtypes in the 2020 WHO classification), and rapid evolution of risk stratification criteria, these tumors exhibit median diagnostic delays of 6 months (9–12). While AI has transformed decision support for prevalent cancers such as breast, lung, and pancreatic malignancies—demonstrating strong alignment with established clinical guidelines—its application to rare, molecularly heterogeneous tumors remains markedly underdeveloped (13–15). No prior study has systematically evaluated LLMs’ capacity to synthesize multimodal sarcoma data or benchmarked their performance against standardized clinical benchmarks. This creates a critical barrier to deploying these tools in settings where specialist expertise is scarce.

This study addresses these gaps by conducting a comprehensive evaluation of ChatGPT and DeepSeek-R1 across 249 bone and soft tissue tumor-related questions, spanning five clinical domains: Clinical Features & Diagnosis, Lab & Imaging Studies, Pathology & Genetics, Staging & Prognosis, and Treatment. We assess both models’ accuracy, error patterns, and performance in single-choice versus multiple-choice formats. Additionally, blinded expert evaluations of multidisciplinary case analyses provide insights into their clinical reasoning capabilities. By synthesizing quantitative metrics with qualitative clinician assessments, this work aims to establish evidence-based guidelines for LLM deployment in orthopedic oncology, while identifying opportunities for model optimization and hybrid AI-human collaboration.

## 2 Methods

### 2.1 Model specifications and configuration

This study evaluated two state-of-the-art large language models: DeepSeek-R1 (version 2.3.1) and OpenAI’s GPT-4o (version gpt-4o-2024-11-20, often unofficially referred to as “4.5.0”). According to OpenAI’s official documentation, GPT-4o is a multimodal model released in May 2024 and is not a variant of GPT-4 Turbo. During the evaluation of both models, real-time web search was enabled to simulate their ability to access the latest information in a real-world clinical setting. DeepSeek-R1 employs a hybrid architecture combining a 280-billion-parameter transformer with a Mixture-of-Experts (MoE) system optimized for medical reasoning, trained on 45 trillion tokens from peer-reviewed oncology literature and clinical guidelines (DeepSeek-AI, 2025). ChatGPT-4o utilizes a 1.8-trillion-parameter dense transformer architecture, fine-tuned via supervised learning and reinforcement learning from human feedback (RLHF) on general-domain corpora (OpenAI, 2025). Both models integrated real-time search engines and multi-step reasoning frameworks to enhance clinical accuracy. We selected ChatGPT-4 (ChatGPT-4o) as the OpenAI comparator because, at the time of data collection (February 2025), it was the most widely accessible, stable, and documented model. This ensured that our evaluation could be reproduced by other researchers under identical conditions. While OpenAI had introduced the o1 reasoning model in late 2024, its preview status, limited access, and variable “reasoning effort” features made it unsuitable for a controlled head-to-head comparison. Therefore, ChatGPT-4o was chosen as a pragmatic, practice-relevant baseline for evaluation.

### 2.2 Question bank design and validation

The study evaluated 249 questions sourced from the Chinese Bone and Soft Tissue Tumor Senior Professional Qualification Examination, categorized into five clinical domains by three board-certified experts: Clinical Features & Diagnosis (n=58), Lab & Imaging Studies (n=46), Pathology & Genetics (n=40), Staging & Prognosis (n=25), and Treatment (n=80). Standard answers were established through expert consensus, with ambiguous items resolved by majority agreement and aligned with the WHO Classification of Bone and Soft Tissue Tumors (2020) and national clinical guidelines. The complete set of 249 questions is provided in the **Supplementary Material** (**Supplementary Material 1**) to ensure reproducibility and facilitate benchmarking in future studies.

### 2.3 Response generation protocol

Questions were presented in English via dedicated interfaces between 2025-02–03 and 2025-02-04. Each question was processed in a new chat session to mitigate session-based learning effects with reset context windows and cleared search caches. Models responded to the standardized prompt: “Please reason based on the questions provided and choose the most appropriate answer. Explain your reasoning in detail, then provide one (single-choice) or multiple (multiple-choice) best answers with justification.”

### 2.4 Multidisciplinary clinical case evaluation

Nine senior clinicians—four bone/soft tissue tumor specialists, two oncologists, one pathologist, one radiologist, and one radiotherapist—evaluated anonymized, randomized responses to an institutional Ewing’s sarcoma case. To prevent bias, evaluators were blinded to model identities and scored seven dimensions on a 0–10 scale: diagnostic accuracy, pathological analysis depth, imaging interpretation, laboratory findings integration, differential diagnosis comprehensiveness, treatment plan feasibility, and multidisciplinary coordination. On this scale, 0 indicated a wholly incorrect or clinically unsafe response, 5 indicated a partially correct or acceptable but incomplete response, and 10 indicated a fully correct, guideline-concordant, and comprehensive response. Intermediate scores reflected gradations between these anchors. For example, in the Diagnostic Accuracy dimension, a score of 0 denoted an entirely incorrect diagnosis, 5 denoted a partially correct but incomplete diagnosis, and 10 denoted a completely accurate diagnosis aligned with expert consensus. Similar anchor definitions were provided for each dimension to ensure rating consistency. This scoring approach is consistent with prior published evaluations of LLMs in oncology (7). Prior to the experts’ scoring, they received unified training on the scoring criteria. Inter-rater reliability analysis of the nine experts’ scoring results showed high consistency (Cohen’s kappa = 0.82, p <.001).

### 2.5 Statistical analysis

Data analysis was performed using SPSS 30.0 (IBM Corp.), and figures and tables were generated using Origin 2024 (OriginLab Corporation). Categorical variables (e.g., accuracy) were compared using the chi-square test. Continuous variables (e.g., expert ratings) were compared using the independent sample t-test. Statistical significance was defined as p < 0.05. Given the multiple comparisons across five clinical domains and two question types, we applied a Bonferroni correction to adjust the significance level. The adjusted significance level was set at p < 0.005 (0.05/10) to control the risk of type I error. All p-values reported in the text are uncorrected, and results that passed this stringent threshold are highlighted in the Discussion.

## 3 Results

### 3.1 Comparative accuracy of ChatGPT and DeepSeek on medical examination questions

**Figure 1:**
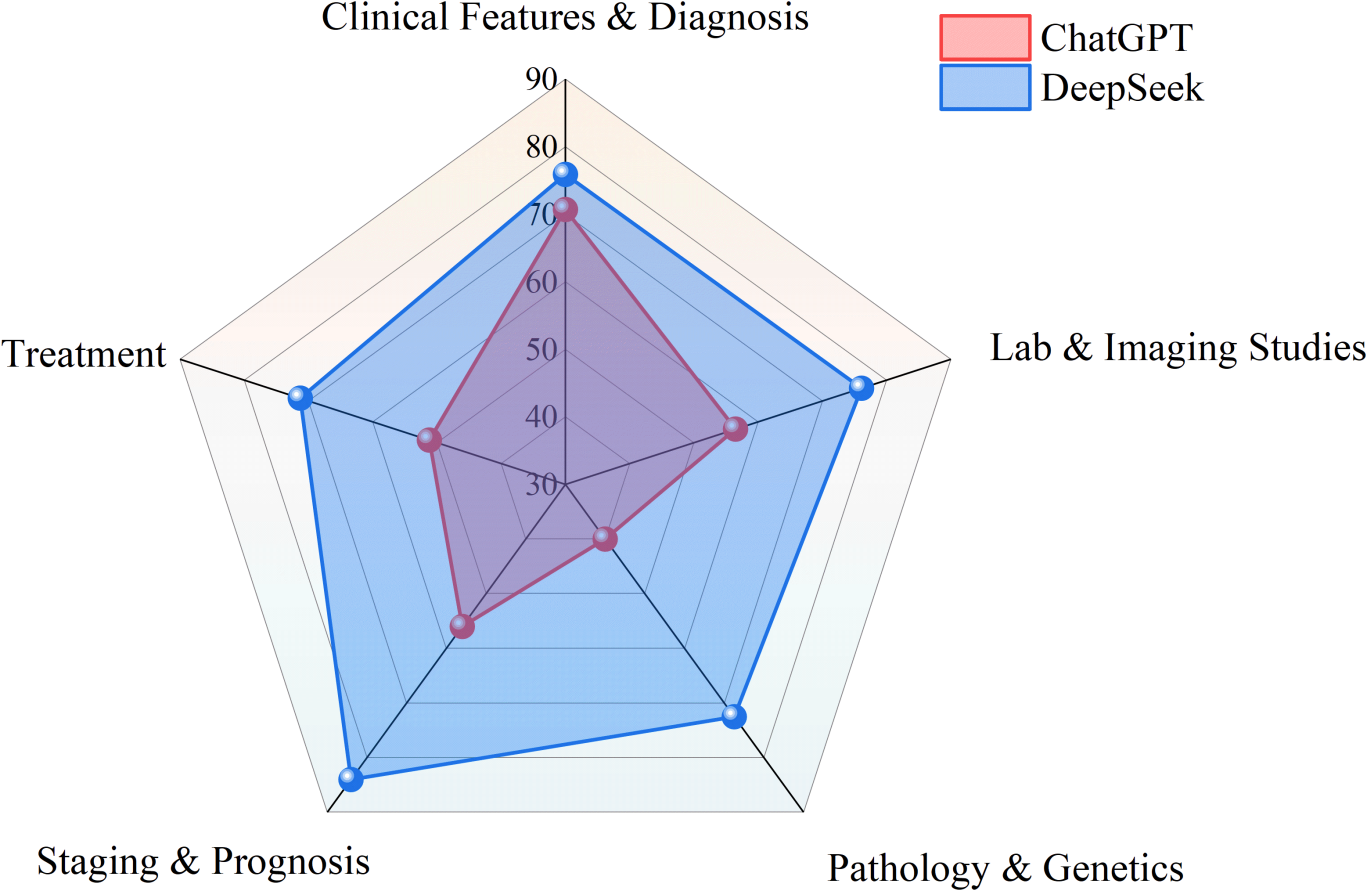
Comparative accuracy of ChatGPT and DeepSeek across question formats.

**Figure 2:**
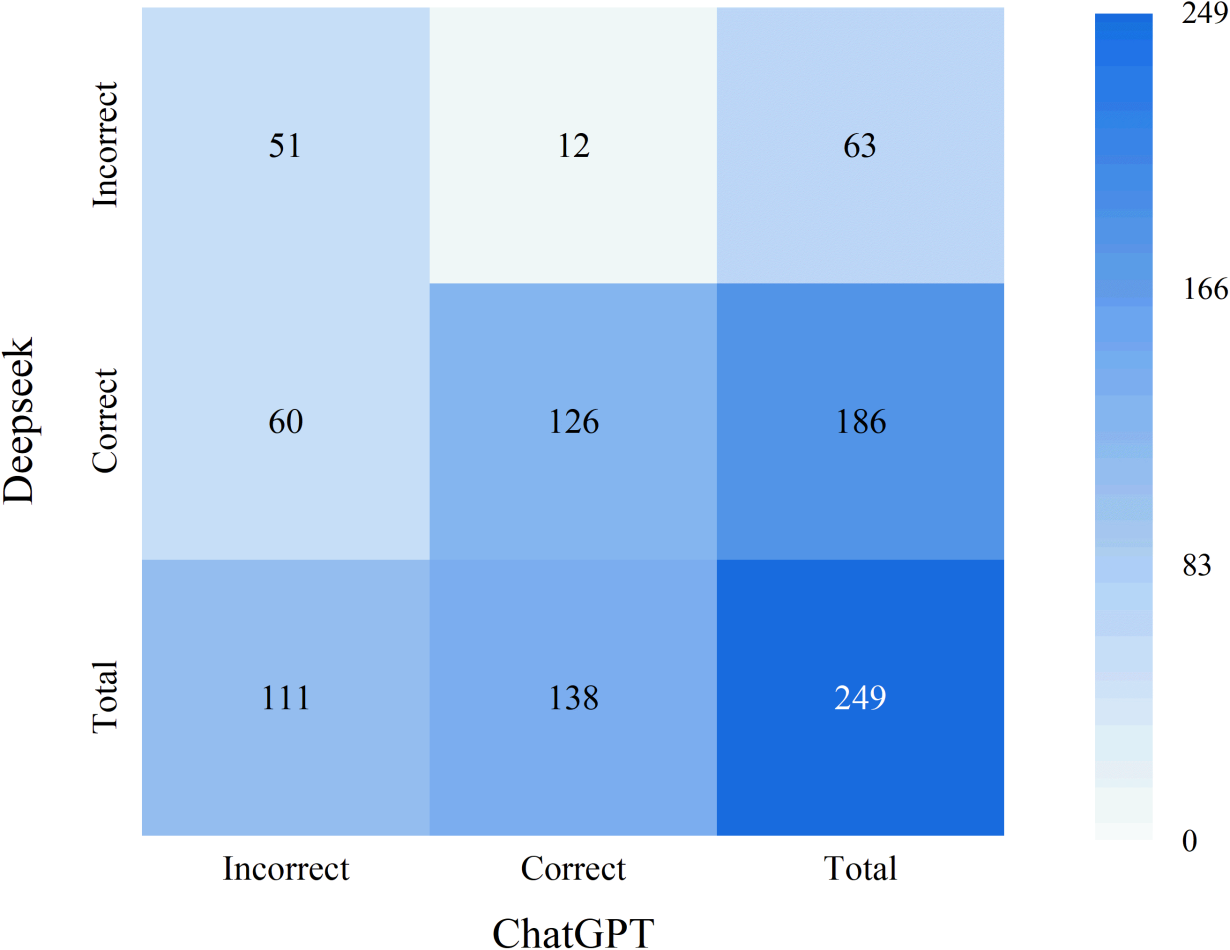
Cross-model accuracy heatmap comparing DeepSeek and ChatGPT responses.

DeepSeek demonstrated statistically significant superiority over ChatGPT on 249 questions related to bone and soft tissue tumors (p < 0.001; **Table 1**). DeepSeek’s accuracy was significantly higher than ChatGPT’s. As shown in **Figure 1a**, DeepSeek’s overall accuracy was 74.7% (186/249), significantly higher than ChatGPT’s 55.4% (138/249). The cross-model accuracy heatmap in **Figure 2** reveals key overlaps and differences: Both models failed on 51 questions (20.5% of the total), while DeepSeek independently answered 60 questions correctly (24.1% of its overall advantage). ChatGPT outperformed DeepSeek on only 12 questions (4.8%).

**Table 1:** Comparative accuracy of ChatGPT and DeepSeek on medical examination questions.

Group	Total	Correct (%)	Wrong (%)	χ²	P
ChatGPT	249	138 (55.4%)	111 (44.6%)	20.35	<0.001
DeepSeek	249	186 (74.7%)	63 (25.3%)		

### 3.2 Performance comparison between ChatGPT and DeepSeek by question type

Significant performance disparities emerged between ChatGPT and DeepSeek across question types (**Table 2**; **Figures 1b, c**). For single-choice questions (SCQ, n = 191), DeepSeek achieved markedly higher accuracy (86.9% [166/191]) compared to ChatGPT (64.9% [124/191]), with a statistically significant difference (χ² = 25.26, p < 0.001). This gap translated to error rates of 13.1% (25/191) for DeepSeek versus 35.1% (67/191) for ChatGPT in SCQs. In multiple-choice questions (MCQ, n = 58), DeepSeek maintained a numerical advantage (34.5% correct [20/58] vs. ChatGPT’s 24.1% [14/58]), though this difference did not reach statistical significance (χ² = 1.50, p = 0.308). Error rates for MCQs were substantial for both models, with ChatGPT producing incorrect answers in 75.9% (44/58) of cases and DeepSeek in 65.5% (38/58).

**Table 2:** Performance comparison between ChatGPT and DeepSeek by question type.

Question type	Group	Total	Correct (%)	Wrong (%)	χ²	P
SCQ	ChatGPT	191	124 (64.9%)	67 (35.1%)	25.26	<0.001
	DeepSeek	191	166 (86.9%)	25 (13.1%)		
MCQ	ChatGPT	58	14 (24.1%)	44 (75.9%)	1.50	0.308
	DeepSeek	58	20 (34.5%)	38 (65.5%)		

SCQ, Single-choice questions; MCQ, Multiple-choice questions.

### 3.3 Performance comparison by knowledge domain in bone and soft tissue tumor management

**Figure 3:**
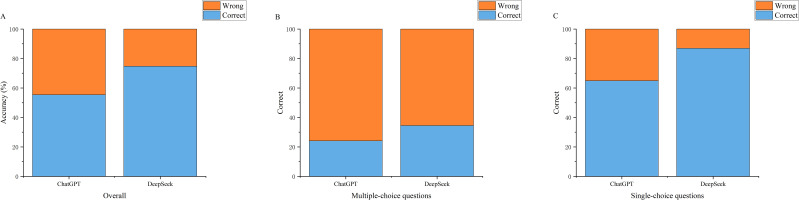
Radar chart comparison of model performance across clinical knowledge domains.

Model performance varied across different clinical knowledge domains (**Table 3**). DeepSeek demonstrated the most significant advantages in Pathology & Genetics and Therapy. The radar charts in **Figure 3** visually demonstrate that DeepSeek maintains its performance advantage across all five evaluation domains. Specifically, DeepSeek demonstrated statistically significantly higher accuracy in Pathology & Genetics (72.5% [29/40] vs. 40.0% [16/40]; χ² = 8.58, p = 0.006) and Therapy (71.3% [57/80] vs. 51.2% [41/80]; χ² = 6.74, p = 0.015).

**Table 3:** Performance comparison by clinical knowledge domain.

Question type	Group	Total	Correct (%)	Wrong (%)	χ²	P
Clinical Features & Diagnosis	ChatGPT	58	41 (70.7%)	17 (29.3%)	0.40	0.675
	DeepSeek	58	44 (75.9%)	14 (24.1%)		
Lab & Imaging Studies	ChatGPT	46	26 (56.5%)	20 (43.5%)	3.94	0.077
	DeepSeek	46	35 (76.1%)	11 (23.9%)		
Pathology & Genetics	ChatGPT	40	16 (40.0%)	24 (60.0%)	8.58	0.006
	DeepSeek	40	29 (72.5%)	11 (27.5%)		
Staging & Prognosis	ChatGPT	25	14 (56.0%)	11 (44.0%)	4.67	0.062
	DeepSeek	25	21 (84.0%)	4 (16.0%)		
Treatment	ChatGPT	80	41 (51.2%)	39 (48.8%)	6.74	0.015
	DeepSeek	80	57 (71.3%)	23 (28.7%)		

### 3.4 Expert evaluation of complex clinical case analysis

Significant performance differences emerged in specific clinical dimensions during blinded expert assessment (**Table 4**). DeepSeek significantly outperformed ChatGPT in both image interpretation and overall case analysis scores. DeepSeek demonstrated superior performance in Imaging Interpretation (7.11 ± 0.60 vs. ChatGPT’s 6.00 ± 0.71; p = 0.002) and achieved a higher overall score (54.11 ± 2.47 vs. 51.56 ± 1.74; p = 0.022). ChatGPT showed comparable capability in Pathological Analysis (8.00 ± 0.71 vs. DeepSeek’s 7.67 ± 0.71; p = 0.332) and Multidisciplinary (MDT) recommendations (7.89 ± 0.78 vs. 7.56 ± 0.53; p = 0.305). No statistically significant differences were observed in Laboratory Findings (DeepSeek: 7.22 ± 0.83 vs. ChatGPT: 6.67 ± 0.71; p = 0.147), Differential Diagnosis (8.11 ± 0.93 vs. 7.78 ± 0.44; p = 0.345), or Treatment planning (7.89 ± 0.78 vs. 7.44 ± 0.53; p = 0.176).

**Table 4:** Comparative expert ratings across clinical analysis dimensions.

Evaluation dimension	ChatGPT	DeepSeek	P
Diagnosis	7.78±0.67	8.56±0.88	0.051
Pathological Analysis	8.00±0.71	7.67±0.71	0.332
Imaging Interpretation	6.00±0.71	7.11±0.60	0.002*
Laboratory Findings	6.67±0.71	7.22±0.83	0.147
Differential Diagnosis	7.78±0.44	8.11±0.93	0.345
Treatment	7.44±0.53	7.89±0.78	0.176
Multidisciplinary (MDT)	7.89±0.78	7.56±0.53	0.305
Overall	51.56±1.74	54.11±2.47	0.022*

The asterisk (*) in Table 4 indicates a statistically significant difference (p < 0.05).

## 4 Discussion

This study establishes DeepSeek’s substantial superiority over ChatGPT in clinical reasoning for bone and soft tissue tumors, achieving a 19.3% higher overall diagnostic accuracy (74.7% vs. 55.4%, p<0.001), a statistically significant margin with critical implications for reducing diagnostic variability in clinical practice. The performance disparity was most pronounced in single-choice questions. DeepSeek outperformed ChatGPT by 22.0% (86.9% vs 64.9%, p<0.001), likely attributable to its specialized architecture’s precision in filtering definitive answers from structured clinical data. Domain-specific analyses further revealed DeepSeek’s advanced capability in synthesizing complex multidisciplinary evidence, particularly in pathology/genetics (+32.5%, p=0.006) and treatment planning (+20.1%, p=0.015)—domains requiring integration of histopathological, genetic, and therapeutic guidelines. Blinded expert evaluations corroborated these findings, with DeepSeek demonstrating superior imaging interpretation (Δ+1.11, p=0.002) and comprehensive case analysis (Δ+2.55, p=0.022), underscoring its utility in scenarios demanding radiological-pathological correlation. DeepSeek uniquely resolved 24.1% of questions where ChatGPT erred, highlighting its enhanced reasoning in ambiguous or data-dense contexts. However, shared errors across both models (20.5% of total questions) persisted, particularly in rare tumor subtype classification, emphasizing inherent limitations in LLM-based systems for low-prevalence conditions. These results position DeepSeek as a robust decision-support tool in orthopedic oncology while advocating for hybrid AI-clinician frameworks to mitigate systematic gaps, ensuring AI augments—rather than replaces—expert judgment in high-stakes diagnostic workflows.

Prior research has extensively documented ChatGPT’s capabilities in general medical tasks, achieving 60–92% accuracy in standardized assessments such as the USMLE (16–18). However, comparative analyses between ChatGPT and domain-optimized models like DeepSeek remain scarce, particularly in rare, molecularly complex tumors. While studies in orthopedics highlight ChatGPT’s role in preoperative planning and imaging analysis, none address the unique challenges of bone and soft tissue tumors—characterized by histopathological mimicry and rapid molecular classification updates (WHO 2020). Our study fills this critical void by providing the first head-to-head evaluation of DeepSeek and ChatGPT in this domain, revealing a 19.3% accuracy gap (74.7% vs. 55.4%, p<0.001) and DeepSeek’s superior performance in integrating histopathological and therapeutic guidelines. These findings align with the emerging emphasis on domain-specific architectures, positioning DeepSeek as a transformative tool for reducing diagnostic delays in resource-limited settings where multidisciplinary expertise is scarce.

Recent studies have begun to evaluate the role of large language models in sarcoma care. Li et al. demonstrated that GPT-4 aligned better with German S3 guidelines for soft tissue sarcoma than GPT-3.5, although both produced misleading or potentially unsafe answers in certain cases (19). Valentini et al. analyzed ChatGPT’s reliability as a patient information source and found wide variability, with many responses being inadequate (20). In the clinical decision-making setting, Ammo et al. assessed ChatGPT-4o in multidisciplinary sarcoma tumor boards, reporting heterogeneous performance across specialties, with strong outputs in surgery but poor reliability in radiation oncology (21). Finally, Li et al. showed that integrating guideline-based retrieval into GPT-4o significantly enhanced its accuracy, comprehensiveness, and safety when answering soft tissue sarcoma queries (22).Taken together, these studies underscore both the promise and the limitations of LLMs in sarcoma research. Unlike prior work that assessed single-model performance or guideline adherence, our study provides the first direct head-to-head comparison between DeepSeek-R1 and ChatGPT-4o across 249 structured clinical questions and an expert-rated case study, thereby offering new insights into relative model strengths and clinical applicability.

The performance advantage of DeepSeek likely stems from two key architectural innovations. First, its advanced reasoning capabilities—a recent breakthrough in clinical AI—enable dynamic synthesis of evolving medical knowledge with case-specific data, a critical requirement in sarcoma diagnostics where guidelines frequently update. Unlike ChatGPT’s reliance on static knowledge recall, DeepSeek demonstrates contextual reasoning to resolve ambiguities, a capability aligned with oncologists’ diagnostic workflows. Second, DeepSeek’s hybrid architecture combines a 280-billion-parameter Transformer with Mixture-of-Experts (MoE) modules specifically optimized for medical reasoning. This design allows selective activation of domain-specific neural pathways—when processing pathology reports, the model prioritizes WHO classification experts, while genetic queries engage mutation analysis subnets. In contrast, ChatGPT’s dense 1.8-trillion-parameter architecture, trained via general reinforcement learning (RLHF), lacks task-specific optimization, leading to suboptimal performance in multidisciplinary tasks requiring simultaneous integration of histopathological, imaging, and genomic data. These architectural distinctions are particularly impactful in treatment planning, where DeepSeek’s MoE system successfully reconciles conflicting guidelines.

This study’s methodological rigor and domain-specific focus offer critical insights into the comparative performance of LLMs in orthopedic oncology. The two-phase evaluation framework—combining a validated clinical question bank with blinded multidisciplinary case analysis—addresses key limitations of prior LLM evaluations. The study ensured clinical relevance and alignment with established diagnostic standards by leveraging 249 questions from the Chinese Bone and Soft Tissue Tumor Senior Professional Qualification Examination. The question bank’s stratification into five clinical domains enabled granular performance analysis, revealing DeepSeek’s superior accuracy in synthesizing histopathological and therapeutic evidence. Furthermore, using session controls—resetting context windows and search caches for each query—mitigated contamination risks. These design choices collectively enhanced the validity of observed performance disparities, particularly DeepSeek’s 19.3% overall accuracy advantage (p<0.001).

While DeepSeek represents a significant advance in specialized oncology AI, its clinical application will require balancing its diagnostic accuracy with the general adaptability of ChatGPT. Future developments should prioritize domain-specific training, real-time knowledge integration, and hybrid human-machine collaboration to maximize patient outcomes without compromising clinician autonomy. As AI advances, its role may shift from decision support to co-pilot, augmenting (but never replacing) the oncologist’s irreplaceable expertise in managing the complexity of rare and heterogeneous tumors.

### 4.1 Limitation

“First, the question bank used in this study was derived from the Chinese professional qualification certification examination, and its content inevitably reflects Chinese clinical practice patterns and guideline preferences. Although core principles of bone oncology (e.g., WHO classification, AJCC staging) are internationally universal, specific treatment strategies (e.g., preference for certain chemotherapy regimens or radiotherapy dosing) may vary regionally. Therefore, the model performance advantages we observed are most directly applicable in the Chinese clinical context. The generalizability of these findings to other healthcare systems (e.g., NCCN or ESMO guideline-driven settings) requires verification through future studies that include more international question banks.”

“Second, our complex case analysis focused solely on Ewing sarcoma. While this case captures the complexity of multidisciplinary decision-making, it is not representative of all bone and soft tissue tumors, particularly rarer subtypes that are more molecularly heterogeneous (e.g., alveolar rhabdomyosarcoma, myxoid liposarcoma, etc.). The model’s performance on these “rare among rare diseases” remains a key area for future evaluation.

“Third, this study evaluated the model’s ability to reason based on text descriptions. In real-world clinical scenarios, imaging images (such as MRI DICOM files) and pathology slides (digital scans of whole slides) contain a wealth of nuanced information that cannot be fully captured in words. Our evaluation framework did not test the model’s ability to directly process this non-textual multimodal data, a common limitation of current large language models and a key area that needs to be addressed in the next generation of multimodal medical AI.”

## 5 Conclusion

This study demonstrates DeepSeek’s superior performance over ChatGPT in bone and soft tissue tumor clinical practice, achieving a 19.3% higher overall accuracy (74.7% vs. 55.4%, p < 0.001) and excelling in specialized domains like pathology/genetics (72.5% vs 40.0%) and treatment planning (71.3% vs 51.2%). The findings validate domain-specific LLMs as valuable tools for augmenting clinical reasoning, particularly in synthesizing histopathological and imaging data. However, shared errors (51/249 questions) and ChatGPT’s comparable performance in pathological analysis highlight persistent challenges in rare tumor subtype diagnosis, necessitating hybrid AI-clinician workflows. Future efforts should prioritize expanding evaluations to rarer tumor subtypes, improving real-world integration with clinical workflows, and addressing limitations in multiple-choice reasoning. By aligning AI development with domain-specific clinical needs, such tools could bridge expertise gaps in resource-limited settings while maintaining clinician oversight for complex cases.

Our conclusions should not be overly interpreted as DeepSeek being universally superior to other models in all clinical scenarios. Its performance requires further validation in key areas: handling a wider range of rare tumor subtypes, integrating real-world imaging and pathology image data, and adapting to diverse international guidelines. Ultimately, the true value of AI models lies in their use as auxiliary tools, collaborating with clinicians in hybrid workflows to improve diagnostic accuracy and the quality of treatment decisions.

## Data Availability

The original contributions presented in the study are included in the article/**Supplementary Material**. Further inquiries can be directed to the corresponding author.
