# Supplementary material for: A comparative study on DeepSeek and ChatGPT for bone and soft tissue tumor clinical practice
Source: Front Oncol. 2026 Jan 9;15:1642880. doi: 10.3389/fonc.2025.1642880 (PMC12827084; doi:10.3389/fonc.2025.1642880)
Supplement: Supplementary file 1 [file DataSheet1.docx]

1. Single-choice questions

The patient is a 17-year-old male with pain, swelling and joint movement disorder below the left knee joint for 2 months. Physical examination: The limb below the left knee joint is 3cm thicker than the opposite side, the surface skin temperature is high, and venous distension and local tenderness can be seen. X-rays show osteolysis and osteoblastic destruction of the left tibial metaphysis, with solar radiation-like periosteal reaction and soft tissue masses. Alkaline phosphatase 485U/L.

The most likely diagnosis among the following is ()

A. Osteomyelitis

B. Giant cell tumor of bone

C. Ewing sarcoma

D. Chondrosarcoma

E. Osteosarcoma

2. Single-choice questions

The patient is a 42-year-old male with dull pain and discomfort in the lower end of the right thigh for 2 years, which gradually worsens and swells. There is a history of right knee joint trauma in the past.

The surgical stage of the disease is ()

A.3

B.ⅠA

C.ⅠB

D.ⅡA

E.ⅡB

3. Single-choice questions

The patient is a 42-year-old male. The main cause is dull pain and discomfort in the lower end of the right thigh for 2 years, which gradually worsens and swells. There is a history of right knee joint trauma in the past.

The most likely disease considered for diagnosis is (prompt CT examination shows expansive destruction of the lower end of the right femur, incomplete bone cortex, and some lesions enter the soft tissue; MRI examination shows T low signal, T mixed signal, and fluid level. Pathological biopsy shows mononuclear stromal cells and multinuclear giant cells.) ()

A. Osteosarcoma

B. Enchondroma

C. Chondrosarcoma

D. Ewing sarcoma

E. Malignant fibrous histiocytoma

4. Single-choice questions

The patient is a 14-year-old female. The lower part of the left thigh was bruised and swollen for 1 month. The symptoms have worsened in the past 2 weeks, which are obvious at night and the body temperature is normal.

The diagnosis currently considered is (the puncture biopsy of the lesion showed that the puncture material was rich in cell components, epithelial or spindle-shaped, of varying sizes, with darkly stained nuclei, active nuclear division phases, and small spindle-shaped or flaky tumor bone-like tissue between cells; MRI showed: in the lower part of the left femur, there was an abnormal signal of about 8.0cm×5.5cm in size, nearly oval, with incomplete cortex.) ()

A. Fracture callus formation of the lower part of the left femur

B. Osteomyelitis of the lower part of the left femur

C. GCT of the lower part of the left femur

D. Chondrosarcoma of the lower part of the left femur

E. OS of the lower part of the left femur

F. Fibrosarcoma of the lower part of the left femur

5. Single-choice questions

The patient is a 14-year-old female. She had swelling and pain in the lower part of her left thigh after a bruise for 1 month. The symptoms have worsened in the past 2 weeks, especially at night, and her body temperature is normal.

Therefore, the initial diagnosis should be checked ()

A. Routine blood test

B. Complete biochemical test

C. Anteroposterior and lateral X-ray of the middle and lower part of the left femur

D. ECT whole body bone scan

E. Routine urine test

6. Single choice questions

The patient is a 14-year-old female. She had swelling and pain in the lower part of her left thigh after a bruise for 1 month. The symptoms have worsened in the past 2 weeks, especially at night, and her body temperature is normal.

The treatment measures to be taken include (the lung CT scan showed (-), and no skip lesions were found in the MR examination of the lower part of the left femur.) ()

A. Use of antibiotics

B. Use of non-steroidal anti-inflammatory drugs

C. Radiotherapy

D. Radionuclide therapy

E. Simple chemotherapy

F. Comprehensive treatment such as neoadjuvant chemotherapy and surgery

7. Single choice questions

Male, 10 years old. Pain and swelling at the lower end of the right thigh, increased skin temperature, and high fever of 39.5℃. Clinical suspicion of acute suppurative osteomyelitis.

The most valuable auxiliary examination is ()

A. X-ray examination

B. CT examination

C. Blood culture

D. Local puncture

E. Routine blood examination

8. Single choice questions

Male, 67 years old, was diagnosed with "thyroid cancer" 3 years ago and underwent surgery. Two months ago, he felt chest and back pain without obvious cause, which did not ease after rest and worsened at night. The painkillers were not effective. The pain gradually worsened in the past half month, making it difficult to sit and stand, and he has been bedridden. Physical examination revealed obvious tenderness in the chest and back, and normal sensation and movement in both lower limbs. However, which of the following examinations is most helpful for diagnosis () A. Thoracic spine X-ray B. Thoracic spine CT + 3D reconstruction C. Thoracic spine MRI D. Isotope bone scan E. Biopsy 9. Single-choice questions The one that does not show expansion changes on the X-ray is () A. Giant cell tumor of bone B. Aneurysmal bone cyst C. Enchondroma D. Typical osteosarcoma E. Osteoblastoma 10. Single-choice questions In the three-step treatment principle for cancer pain, "on-time administration" refers to the drug's () A. Half-life B. Action time C. Drug resistance D. Effective period E. Analgesic effect 11. Single-choice questions

According to the three-step medication principle, the medication suitable for moderate cancer pain is ()

A. Codeine, buprenorphine, ibuprofen

B. Hydromorphone, dextropropoxyphene, andorfen

C. Dihydrocodeine, indomethacin, methadone

D. Codeine, codeine aminophenazone, pentazocine

E. Fentanyl transdermal patch, tramadol, and chimantin

12. Single-choice questions

The lesion with pathological changes similar to osteoid osteoma is ()

A. Osteoblastoma

B. Intracortical osteosarcoma

C. Chronic sclerosing osteomyelitis

D. Non-ossifying fibroma

E. Fibrous cortical defect

13. Single-choice questions

The medication principle that does not conform to the three-step analgesic treatment plan is ()

A. Medication according to the step

B. Medication when in pain

C. Individualized medication

D. Oral medication as much as possible

E. Administer medication on time

14. Single-choice questions

The following is not a benign self-healing type ()

A. fibrous cortical defect

B. solitary bone cyst

C. giant cell tumor of bone

D. exophytic chondroma

E. endochondroma

15. Single-choice question

Physical examination revealed round back deformity, spinal X-ray found bamboo-like changes, and sacroiliac joint fusion. Suspected ()

A. Spinal tuberculosis

B. Ankylosing spondylitis

C. Intervertebral disc herniation

D. Spinal stenosis

E. Eosinophilic granuloma

16. Single-choice questions

For fibroblastic fibroma, the following statement is incorrect ()

A. The clinical manifestations are not specific and can be easily misdiagnosed

B. This disease is a benign tumor, but it is highly invasive

C. The main treatment method is surgical resection

D. Radiotherapy or chemotherapy alone is effective for tumors

E. Local curettage has a high recurrence rate

17. Single-choice questions

The surgery for curettage of giant cell tumor of distal femur is ()

A. Radical resection

B. Intracapsular resection

C. Extensive curettage

D. Extensive resection

E. Marginal resection

18. Single-choice questions

For the qualitative diagnosis of osteosarcoma, the following description is incorrect ()

A. It requires a combination of clinical, imaging, and pathology

B. MR examination is more valuable than traditional imaging

C. MR has obvious advantages in showing the scope of intramedullary invasion and skip lesions

D. Radionuclide scanning can show skip lesions in the bone

E. CT can show bone destruction and the relationship between lesions and surrounding tissues

19. Single-choice questions

For the diagnosis and efficacy judgment of bone and joint tuberculosis, the most commonly used is ()

A. Systemic symptoms

B. Local signs

C. X-ray examination

D. Laboratory examination

E. CT examination

20. Single-choice questions

The incorrect description of radiotherapy for bone and soft tissue tumors is ()

A. Radiotherapy for soft tissue sarcoma includes postoperative radiotherapy, preoperative radiotherapy and after loading brachytherapy

B. Radiotherapy has adverse effects on children's bone growth

C. Most bone and soft tissue tumors are sensitive to radiotherapy

D. Radiotherapy can cause bone necrosis

E. Radiotherapy has a certain limit on dose

21. Single-choice question

The soft tissue sarcoma most sensitive to chemotherapy is ()

A. Childhood rhabdomyosarcoma

B. Fibrosarcoma

C. Liposarcoma

D. Synovial sarcoma

E. Epithelioid sarcoma

22. Single-choice question

For Enneking staging, the stage that osteosarcoma cannot belong to is ()

A. Stage 3

B. Stage IA

C. Stage IB

D. Stage II

E. Stage III

23. Single-choice question

For osteochondroma, the following statement is incorrect ()

A. The malignant rate of single-episode is lower than that of multiple-episode

B. The fibrous perichondrium on the surface of the tumor is continuous with the surrounding normal periosteum

C. The thickness of the cartilage cap rarely exceeds 2cm

D. The cortical bone of the tumor is continuous with the host bone

E. The actual size of the tumor is smaller than that shown on the X-ray film

24. Single-choice question

For chondromyxoid fibroma, the following statements are correct ()

A. It is the most common benign cartilage tumor

B. It mostly occurs in middle-aged and elderly people

C. Radiotherapy is sensitive to this disease

D. Chemotherapy can be used as an adjuvant treatment

E. The main treatment for this disease is surgical resection

25. Single-choice questions

The cause of most Ewing sarcomas is ()

A. Gene deletion

B. Translocation of chromosomes 8 and 12

C. Translocation of chromosomes 11 and 21

D. Chromosome translocation forms the EWS/ERG fusion gene

E. Chromosome translocation forms the EWS/Fli1 fusion gene

26. Single-choice questions

The treatment method for enchondroma occurring in the phalanges is ()

A. Resection of the phalanges to completely eliminate the lesion

B. Tumor scraping + bone grafting

C. Tumor margin resection

D. Tumor scraping and bone grafting + chemotherapy

E. Tumor scraping and bone grafting + radiotherapy

27. Single-choice questions

The main application of radionuclide bone scanning in bone and joint tumors does not include ()

A. Bone imaging can use its high sensitivity to screen bone metastases early

B. According to the concentration of radionuclide, assist in the diagnosis of benign and malignant bone tumors

C. PET-CT can help find the primary tumor, distinguish benign and malignant tumors or lesions, and assist in clinical tumor staging

D. Determine whether limb-saving surgery can be performed based on the bone scan performance

E. Monitor tumor recurrence after surgery

28. Single-choice questions

The X-ray manifestations of giant cell tumor of bone do not include ()

A. Expansibility

B. Eccentricity

C. Soap bubble-like

D. Can invade joints

E. Often involve the backbone

29. Single-choice questions

The most basic and pathological diagnostic feature of osteosarcoma is ()

A. Obvious tumor cell atypia

B. Deep and tight nuclei, nuclear division

C. Tumor cells directly produce bone-like tissue

D. Tumor cell diversity

E. Fibroblastic osteosarcoma is morphologically similar to malignant fibrous histiocytoma

30. Single-choice questions

The surgical indications for osteochondroma do not include ()

A. Huge tumors affect appearance

B. Tumors are significantly enlarged and suspected of malignant transformation

C. Tumors grow in the metaphysis

D. Tumors affect joint function

E. Tumors produce compression symptoms

31. Single-choice questions

The differential diagnosis of bone schwannoma and malignant schwannoma does not include ()

A. Clinical symptoms of schwannoma are mild, and pain and neurological symptoms are rare

B. Malignant schwannoma also has pain at rest

C. Imaging manifestations are very helpful in distinguishing the two

D. Malignant schwannoma often has muscle weakness and sensory impairment

E. Mild cell atypia and nuclear division images can be seen in schwannoma

32. Single-choice questions

The common complications of bone and joint tuberculosis do not include ()

A. Sinus formation

B. Pathological dislocation or subluxation of joints

C. Joint fusion

D. Joint deformity or ankylosis

E. Limb shortening

33. Single-choice questions

Which of the following is incorrect about the preoperative preparation for bone and joint tuberculosis ()

A. Conduct systemic anti-tuberculosis treatment for 2 to 4 weeks

B. Correct anemia and hypoproteinemia

C. Control mixed infection first and then perform lesion removal

D. Provide sufficient calories, protein and vitamins

E. Cure pulmonary tuberculosis first and then perform lesion removal

34. Single-choice questions

The most common way of metastasis of bone metastasis is ()

A. Blood and lymphatic metastasis

B. Jump metastasis

C. Direct dissemination

D. Blood metastasis

E. Lymphatic metastasis

35. Single-choice questions Regarding long bone ameloblastoma, the following statement is correct ()

A. Long bone ameloblastoma is a primary benign tumor of bone

B. Long bone ameloblastoma is generally not believed to originate from epithelial cells

C. Long bone ameloblastoma is prone to occur in the diaphysis and epiphysis of the tibia

D. X-ray manifestations are often osteoblastic

E. Long bone ameloblastoma does not need to be differentiated from fibrous dysplasia

36, Single choice questions

Regarding bone neurofibroma, the following statement is incorrect ()

A. The tumor is composed of Schwann cells, neural fascia cells and fibroblasts

B. Degeneration, cystic change and hemorrhage in the tumor are rare

C. Single bone neurofibroma and bone nerve sheath tumor are difficult to distinguish in diagnosis

D. MRI may show "target sign"

E. Bone neurofibroma often occurs in children

37, Single choice questions

Which of the following statements about bone hemangioma is correct ()

A. Bone hemangioma is a benign tumor with a capsule visible to the naked eye

B. Spinal hemangioma can affect the entire spine

C. Long bone hemangioma is rare and often occurs in the bone shaft

D. Spinal hemangioma is generally difficult to diagnose

E. Bone hemangioma does not need to be differentiated from osteoporosis in the diagnosis

38. Single-choice questions

Which of the following statements about bone vascular tumors is wrong ()

A. Not all bone vascular tumors are true tumors

B. The three major symptoms of glomus tumors are intermittent pain, tenderness, and cold sensitivity

C. Bone hemangioendothelioma is a malignant tumor that can metastasize

D. Bone hemangiopericytoma has a better prognosis than angiosarcoma

E. Angiosarcoma is a highly malignant tumor originating from vascular endothelial cells or their precursor cells

39. Single-choice questions

Which of the following statements about bone lipoma and liposarcoma is wrong? ()

A. Bone lipoma is often found in the epiphysis of long tubular bones, most commonly in the intertrochanteric and subtrochanteric regions of the proximal femur

B. X-rays of intraosseous lipomas show round osteolytic lesions, with focal calcification and residual trabeculae

C. X-rays of bone liposarcoma can reveal scattered calcification spots and residual trabeculae in the tumor

D. The most common site of bone liposarcoma is the epiphysis of long tubular bones

E. MRI examinations of bone lipomas show high signals in both T1 and T2 weighted images, which are significantly enhanced after enhanced scanning

40. Single-choice questions

Which of the following statements about the common sites of bone tumors is incorrect? ()

A. Giant cell tumors of bone are always located at the epiphysis and proximal bone ends

B. Cartilaginous tumors are always located at the epiphysis or at the bone ends that connect or span the growing cartilage

C. Cartilaginous tumors are common in the skull

D. Chordomas almost always occur at the skull base, sacrum or spine, and are extremely rare in the limbs

E. Ameloblastomas often occur at the tibia or ulna

41. Single-choice question

Regarding the growth barrier and mechanism of bone tumors, the one that is not a natural barrier is ()

A. Cortical bone

B. Articular cartilage

C. Fascial septa, myometrial septa and ligaments

D. Joint capsule, tendon sheath

E. Muscle

42. Single-choice question

Regarding the clinical characteristics of acute hematogenous osteomyelitis, the following is incorrect ()

A. Common in children under 12 years old

B. Mostly occurs in the epiphyseal end of long bones

C. The most common pathogen is Streptococcus

D. Early diagnosis mainly relies on local layered puncture

E. X-ray examination generally shows bone destruction and periosteal reaction about 2 weeks after onset

43. Single-choice question

Regarding spinal tuberculosis complicated with paraplegia, the following is incorrect ()

A. The incidence of paraplegia in spinal tuberculosis is about 10%

B. Thoracic tuberculosis is more common with paraplegia

C. Spinal appendage tuberculosis is rare, but once it occurs, it is prone to paraplegia

D. Paraplegia caused by cervical, cervicothoracic and thoracolumbar tuberculosis is less common than thoracic tuberculosis

E. Lumbar tuberculosis is prone to compression of the cauda equina

44, Single-choice questions

Regarding benign active tumors, the correct answer is ()

A. Includes all giant cell tumors of bone

B. Generally does not cause symptoms

C. Often causes pathological fractures

D. X-ray films show no obvious boundary between the tumor and the surrounding normal tissues

E. Surgery is generally performed by intracapsular resection

45, Single-choice questions

Which of the following statements is correct about the treatment of fibrosarcoma ()

A. The main treatment method is surgical resection

B. The main treatment method is surgery + radiotherapy

C. The main treatment method is chemotherapy + radiotherapy

D. Radiotherapy and chemotherapy are not sensitive to this disease

E. The main treatment methods are chemotherapy, surgery, and radiotherapy

46. Single-choice questions

Which of the following is incorrect about the clinical characteristics of central vertebral tuberculosis ()

A. More common in children under 10 years old

B. Common in the lumbar spine

C. Rapid progression of the lesion

D. Generally only one vertebra is invaded

E. The entire vertebra is often compressed into a wedge shape

47. Single-choice questions

The commonly used staging method for musculoskeletal system tumors internationally is ()

A. Enneking staging system

B. TNM staging

C. Kawaguchi staging

D. Pathological staging

E. Clinical staging

48. Single-choice questions

The patient is a 12-year-old boy with left calf pain for 1 month, which is progressively aggravated and accompanied by fever. Physical examination: The upper end of the tibia of the affected leg is swollen with tenderness and increased skin temperature. X-ray shows: end-shaped osteolytic destruction of the upper tibia. For diagnosis, first consider ()

A. Osteosarcoma

B. Ewings sarcoma

C. Chondrosarcoma

D. Giant cell tumor of bone

E. Osteochondroma

49. Single choice questions

The patient is a 12-year-old boy with left calf pain for 1 month, which is progressively aggravated and accompanied by fever. Physical examination: The upper end of the tibia of the affected leg is swollen with tenderness and increased skin temperature. X-ray shows: end-shaped osteolytic destruction of the upper tibia. The most likely laboratory test results are ()

A. Codman triangle on X-ray

B. Inflammatory cell infiltration on puncture biopsy

C. MRI shows low signal on TWI and TWI of lesions

D. Onion skin-like reaction on X-ray

E. Lung CT shows multiple nodules in the lungs

50. Single-choice questions

The patient is a 12-year-old boy with left calf pain for 1 month, which is progressively aggravated and accompanied by fever. Physical examination: swelling and tenderness at the upper end of the tibia of the affected leg, and increased skin temperature. X-ray shows: punctate osteolytic destruction at the upper end of the tibia. The most likely indicator of a poor prognosis is ()

A. Weight loss in the child

B. Fever > 39℃

C. The mass does not shrink after chemotherapy

D. Multiple metastases in the lungs

E. Contamination of surgical margins

51. Single-choice questions

The patient is a 10-year-old boy who complained of repeated pain in the middle and lower part of the right calf for half a year. Physical examination: There is no obvious swelling in the middle and lower part of the right calf, severe tenderness, and normal ankle joint movement. Imaging shows that there is an eccentric osteolytic destruction of about 1.5cm×2.5cm in the right tibial metaphysis, with visible sclerotic edges and intact cortex. The most likely diagnosis is ()

A. Ossifying fibroma

B. Benign fibrous histiocytoma

C. Non-ossifying fibroma

D. Dysplastic fibers

E. Giant cell tumor of bone

52. Single choice questions

The patient is a 10-year-old male who complained of repeated pain in the middle and lower part of the right calf for half a year. Physical examination: There is no obvious swelling in the middle and lower part of the right calf, severe tenderness, and normal ankle joint movement. Imaging shows that there is an eccentric osteolytic destruction of about 1.5cm×2.5cm in the right tibial metaphysis, with visible sclerotic edges and intact cortex. The current treatment for this patient should be ()

A. Close observation, surgical treatment if necessary

B. Immediate radical surgical resection

C. Immediate surgery combined with radiotherapy

D. Immediate chemotherapy followed by surgical treatment

E. Immediate chemotherapy + surgery + radiotherapy

53. Single-choice question

The patient is a male, 11 years old, diagnosed with stage ⅡB osteosarcoma of the distal femur before surgery. The next treatment measure is ()

A. Regular review and observation of changes

B. Local radiotherapy

C. Reoperation, at least to a wide margin

D. Chemotherapy after wound healing

E. Amputation

54. Single-choice question

The patient is a male, 11 years old, diagnosed with stage ⅡB osteosarcoma of the distal femur before surgery. The surgery involves segmental tumor resection and artificial joint replacement in the normal tissue outside the reaction zone. The surgical boundary belongs to ()

A. Intracapsular surgery

B. Marginal surgery

C. Extensive surgery

D. Radical surgery

E. Contaminated radical surgery

55. Single-choice question

The patient is a male, 11 years old, diagnosed with stage ⅡB osteosarcoma of the distal femur before surgery. Postoperative pathology report: Tumor cells can be seen at the edge of the resection, so its surgical boundary belongs to ()

A. Intracapsular surgery

B. Marginal surgery

C. Extensive surgery

D. Radical surgery

E. Contaminated radical surgery

56. Single-choice questions

The patient is a 12-year-old male. He has an eccentric osteolytic lesion at the epiphysis of the left tibia, with unclear boundaries, intact cortical bone, and no periosteal reaction. The most likely diagnosis is ()

A. Non-ossifying fibroma

B. Chondroblastoma

C. Giant cell tumor of bone

D. Aneurysmal bone cyst

E. Osteoblastoma

57. Single-choice questions

The patient is a 12-year-old male. The main reason is a collision while playing basketball at school, sudden left humeral pain, and fear of movement and hospitalization. There is no previous medical history. The best emergency treatment at this time is ()

A. puncture biopsy

B. emergency surgery, tumor curettage, bone graft internal fixation

C. small splint or plaster external fixation

D. high-dose chemotherapy

E. radiotherapy

F. emergency surgery, tumor segment resection, prosthesis replacement

58. Single-choice questions

The patient is a 12-year-old boy. The main reason is a collision while playing basketball at school, sudden left humeral pain, and he was afraid to move and was admitted to the hospital. There is no previous medical history. The items that should be urgently checked for a clear diagnosis are (prompt physical examination: stable vital signs, left humeral shaft swelling, no deformity, obvious tenderness, normal left hand movement, normal radial artery pulsation.) ()

A. blood routine

B. whole body bone scan

C. blood biochemistry

D. left humeral MRI or CT

E. left humeral X-ray film

F. abdominal X-ray film

59. Single-choice questions

The patient is a 12-year-old boy. The main reason was a collision while playing basketball at school, which caused sudden pain in the left humerus. He was afraid to move and was admitted to the hospital. He had no previous medical history. The most likely diagnosis was (the X-ray of the left humeral metaphysis showed an oblique fracture without displacement, and the cortex of the metaphysis was thinned and expanded, with an osteolytic lesion, no calcification in the lesion, and no periosteal reaction.) ()

A. Osteosarcoma with pathological fracture

B. Ewing sarcoma with pathological fracture

C. Bone cyst with pathological fracture

D. Lymphoma with pathological fracture

E. Giant cell tumor of bone with pathological fracture

F. Dysplasia of fibrous structure with pathological fracture

60. Single-choice questions

The patient was a 13-year-old male who complained of proximal right thigh pain, which was more obvious at night. Oral indomethacin relieved the symptoms. X-rays showed that the cortex of the middle and upper parts of the right femur was spindle-shaped, dense and hypertrophic, with no obvious periosteal reaction. It seems that the most likely diagnosis is ()

A. Non-ossifying fibroma

B. Osteosarcoma

C. Osteoid osteoma

D. Osteoblastoma

E. Chronic sclerosing osteomyelitis

61. Single-choice question

The patient, a 13-year-old male, complained of right thigh proximal pain, which was more obvious at night. Oral indomethacin relieved the symptoms. X-ray showed that the cortex of the middle and upper part of the right femur was spindle-shaped, dense and hypertrophic, and no obvious periosteal reaction was observed. The most effective laboratory test for a clear diagnosis is ()

A. Ultrasound examination

B. CT bone window thin-layer scan

C. Conventional CT scan + enhancement

D. DSA examination

E. Radionuclide bone scan

62. Single-choice question

The patient, a 14-year-old male, had pain, swelling and joint movement disorders above the left knee joint. Physical examination: The limb above the left knee joint was 4cm thicker than the contralateral side, with high surface skin temperature, venous distension and local tenderness. X-ray showed osteolysis and osteoblastic destruction of the left femoral metaphysis, with periosteal reaction and soft tissue mass. Alkaline phosphatase 355U/L. An examination that must be performed on this patient ()

A. Blood routine

B. CT

C. MRI

D. Bone scan

E. Pathological biopsy

63. Single-choice question

The patient is a 14-year-old male with pain, swelling and joint movement disorder above the left knee joint. Physical examination: The limb above the left knee joint is 4cm thicker than the opposite side, with high surface skin temperature, venous distension and local tenderness. X-ray shows osteolysis and osteoblastic destruction of the left femoral metaphysis, with periosteal reaction and soft tissue mass. Alkaline phosphatase 355U/L. The most likely diagnosis is ()

A. Osteomyelitis

B. Giant cell tumor of bone

C. Osteosarcoma

D. Chondrosarcoma

E. Ewing sarcoma

64. Single-choice question

The patient is a 14-year-old male with pain, swelling and joint movement disorder above the left knee joint. Physical examination: The limb above the left knee joint is 4cm thicker than the opposite side, with high surface skin temperature, venous distension and local tenderness. X-ray film showed osteolysis and osteoblastic destruction of the left femoral metaphysis, periosteal reaction and soft tissue mass. Alkaline phosphatase 355U/L. If the patient needs surgical treatment, he cannot choose ()

A. En bloc resection of the lesion

B. Lesion curettage and bone grafting internal fixation

C. En bloc resection of the lesion and artificial joint replacement

D. En bloc resection of the lesion and joint fusion

E. Amputation treatment

65. Single-choice question

The patient is a 15-year-old male with swollen left knee for 2 months, which is worse at night. Physical examination: swelling on the upper medial side of the left knee, engorged veins, hard and fixed swelling, tenderness (+), local osteolysis destruction and needle-like periosteal reaction on the X-ray film. The possible diagnosis is ()

A. Bone tuberculosis

B. Osteosarcoma

C. Giant cell tumor of bone

D. Ewing sarcoma

E. Chondrosarcoma

66. Single-choice question

The patient is a 15-year-old male with swollen left knee for 2 months, which is worse at night. Physical examination: swelling of the left upper medial knee, engorged veins, hard and fixed swelling, tenderness (+), local osteolytic destruction and spiculate periosteal reaction on X-ray. The feasible diagnosis method is ()

A. Intraoperative freezing

B. Needle biopsy

C. Small incision biopsy

D. Radionuclide scanning

E. CT scan

67. Single choice questions

The patient is a 15-year-old male with left knee swelling for 2 months, which is worse at night. Physical examination: swelling of the left upper medial knee, engorged veins, hard and fixed swelling, tenderness (+), local osteolytic destruction and spiculate periosteal reaction on X-ray. To clarify the surrounding soft tissue and blood vessel conditions, the best examination is ()

A. X-ray

B. CT scan

C. Magnetic resonance imaging

D. Radionuclide scanning

E. Soft tissue ultrasound

68. Single choice questions

The patient is a 18-year-old male who was diagnosed with pain in the distal left thigh and a lump for 4 months. Physical examination: Swelling of the distal left thigh, no redness of the skin, no superficial varicose veins, and higher skin temperature than the healthy side. A mass can be palpated on the medial side of the knee, which is hard, tender (+), and fixed. Knee joint swelling, floating patella test (+), limited flexion. X-ray shows: mixed osteolytic and osteoblastic changes in the distal femoral epiphysis, bone destruction, no expansion, Codman triangle, and soft tissue mass shadow can be seen on the medial side of the lower femur. The most likely preliminary diagnosis for this patient is ()

A. Giant cell tumor of bone

B. Osteosarcoma

C. Chondrosarcoma

D. Myeloma

E. Desmoid tumor

69, Single choice questions

The patient is a male, 18 years old, who was diagnosed with pain in the distal left thigh and a mass found 4 months ago. Physical examination: Swelling of the distal left thigh, no redness of the skin, no superficial varicose veins, and higher skin temperature than the healthy side. A mass can be palpated on the medial side of the knee, which is hard, tender (+), and fixed. Knee joint swelling, floating patella test (+), limited flexion. X-ray film showed: mixed osteolytic and osteoblastic changes in the distal femoral metaphysis, bone destruction, no expansion, Codman triangle, and soft tissue mass shadow on the medial side of the lower femur. Pathological examination results showed osteosarcoma, and no metastatic lesions were found in other parts. According to the Enneking staging system, the patient's stage is ()

A.ⅡA

B.ⅡB

C.ⅢA

D.ⅢB

E.ⅠB

70, Single choice questions

The patient is a male, 18 years old, who was diagnosed with pain in the distal left thigh and a mass 4 months ago. Physical examination: Swelling of the distal left thigh, no redness of the skin, no superficial varicose veins, and higher skin temperature than the healthy side. A mass can be palpated on the medial side of the knee, which is hard, tender (+), and fixed. Knee joint swelling, floating patella test (+), limited flexion. X-ray film shows: mixed osteolysis and osteoblastic changes in the distal femoral epiphysis, bone destruction, no expansion, Codman triangle, and soft tissue mass shadow can be seen on the inner side of the lower end of the femur. The best treatment plan for this patient is ()

A. Immediate thigh amputation

B. Preoperative chemotherapy-surgery-postoperative chemotherapy

C. Chemotherapy, if the chemotherapy effect is good, then no surgery

D. Local radiotherapy, supplemented by systemic chemotherapy

E. Immediate limb-saving surgery

71. Single-choice questions

The patient is a male, 18 years old, who was diagnosed with pain in the distal left thigh and a mass 4 months ago. Physical examination: Swelling of the distal left thigh, no redness of the skin, no superficial varicose veins, and higher skin temperature than the healthy side. A mass can be palpated on the inner side of the knee, which is hard, tender (+), and fixed. Knee joint swelling, floating patella test (+), limited flexion. X-ray film showed: mixed osteolysis and osteoblastic changes in the distal femoral epiphysis, bone destruction, no expansion, Codman triangle, and soft tissue mass shadow on the inner side of the lower femur. The patient eventually underwent a thigh amputation due to lack of limb salvage conditions, but 8 months after the operation, he developed left chest pain and cough. Chest X-ray showed a metastatic tumor in the left upper lobe of the lung. Analysis showed that its metastatic pathway may be ()

A. Direct spread

B. Spread through arteries

C. Through venous return

D. Through lymphatic return

E. Spread along the nerve trunk

72. Single-choice questions

The patient was a male, 18 years old, and was diagnosed with pain in the distal left thigh and a mass found 4 months ago. Physical examination: Swelling of the distal left thigh, no redness of the skin, no superficial varicose veins, and higher skin temperature than the healthy side. A mass can be palpated on the inner side of the knee, which is hard, tender (+), and fixed. Knee joint swelling, floating patella test (+), limited flexion. X-ray film shows: mixed osteolysis and osteoblastic changes in the distal femoral epiphysis, bone destruction, no expansion, Codman triangle, and soft tissue mass shadow can be seen on the inner side of the lower end of the femur. Which of the following examinations is not necessary ()

A. Chest X-ray film

B. CT scan of the affected limb

C. Knee arthroscopy

D. MRI of the affected limb

E. Puncture biopsy

73. Single-choice question

The patient is a 22-year-old male. The main cause is pain and swelling in the lower right thigh for 1 month. The necessary follow-up treatment is (indicating that the patient has recovered well after surgery, and the chemotherapy tumor necrosis rate is graded as grade III.) ()

A. Functional exercise and close follow-up are sufficient

B. Change chemotherapy regimen after surgery

C. Continue preoperative chemotherapy for at least 3 to 4 courses

D. Local radiotherapy

E. Immunotherapy

74. Single-choice question

The patient is a 32-year-old male. He came to our hospital for treatment due to right upper limb dysfunction caused by a motorcycle accident for 4 months. Physical examination showed that the shoulder abduction and elbow flexion were incapable, the clavicular part of the pectoralis major was paralyzed, the latissimus dorsi muscle strength was basically normal, and the elbow below the elbow could move. The surgical methods that can be taken for this patient do not include ()

A. Phrenic nerve transfer

B. Accessory nerve transfer

C. Ipsilateral C nerve transfer

D. Oberlin operation

E. Brachialis muscle branch transfer

75. Single choice questions

The patient is a 32-year-old male who came to our hospital for treatment due to a motorcycle accident that caused right upper limb dysfunction for 4 months. Physical examination showed that the shoulder abduction and elbow flexion were incapable, the clavicular part of the pectoralis major was paralyzed, the latissimus dorsi muscle strength was basically normal, and the elbow below the elbow could move. The most likely diagnosis for this patient is ()

A. Total brachial plexus root avulsion

B. Brachial plexus branch injury

C. C5, C6 nerve root injury

D. C5, C6, C7 nerve root injury

E. C8, T1 root injury

76, Single choice question

The patient, male, 32 years old, came to our hospital for treatment due to right upper limb dysfunction caused by a motorcycle accident for 4 months. Physical examination showed that the shoulder abduction and elbow flexion could not be achieved, the clavicular part of the pectoralis major was paralyzed, the latissimus dorsi muscle strength was basically normal, and the movement below the elbow was possible. If an Oberlin operation is performed, the ulnar nerve should theoretically be selected ()

A. Intrinsic branch of the hand

B. Sensory branch

C. Ulnar flexor carpi branch

D. Deep flexor branch of the digitorum

E. Intrinsic branch of the hand or sensory branch

77, Single choice question

The patient, male, 35 years old, was admitted to our hospital due to right calf pain for 2 weeks. Physical examination: The right tibia is slightly bent forward, the upper right leg is locally raised on the anterolateral side, and a mass can be touched, about 4cm×2cm, hard, fixed, slightly tender, no varicose superficial veins, and no abnormal limb sensation and muscle strength. X-ray shows an osteolytic lesion on the anterolateral side of the right tibia, with clear boundaries, surrounding bone sclerosis, serrated bone cortical destruction, and no soft tissue mass. The next step of diagnosis and treatment for this patient usually does not include ()

A. Angiography

B. CT

C. MRI

D. ECT

E. Puncture biopsy

78. Single-choice question

The patient is a 35-year-old male and was admitted to the hospital due to right calf pain for 2 weeks. Physical examination: The right tibia is slightly bent forward, the upper right leg is locally raised on the anterolateral side, and a mass can be touched, about 4cm×2cm, hard, fixed, slightly tender, no varicose superficial veins, and no abnormal limb sensation and muscle strength. X-ray film showed an osteolytic lesion on the anterolateral side of the right tibia, with clear boundaries, bone sclerosis around it, serrated cortical bone destruction, and no soft tissue mass. The patient was diagnosed with ameloblastoma before surgery, and the best treatment method is ()

A. Radiotherapy

B. Chemotherapy

C. Lesion scraping and bone grafting

D. Wide tumor resection and bone grafting

E. Radical amputation

79. Single-choice question The patient was a 35-year-old male and was admitted to the hospital due to right calf pain for 2 weeks. Physical examination: The right tibia was slightly bent forward, and the upper part of the right calf was locally raised on the anterolateral side. A mass of about 4cm×2cm could be touched, which was hard, fixed, and slightly tender. There was no varicose superficial vein, and there was no abnormality in limb sensation and muscle strength. X-ray film showed an osteolytic lesion on the anterolateral side of the right tibia, with clear boundaries, bone sclerosis around it, serrated cortical bone destruction, and no soft tissue mass. The lesions that need differential diagnosis do not include ()

A. Bone fibrous dysplasia

B. Osteomyelitis

C. Non-ossifying fibroma

D. Ameloblastoma

E. Giant cell tumor of bone

80. Single choice question

A 40-year-old male patient had back pain for 2 years. X-ray examination showed that the density of the T10 vertebra was reduced, the trabeculae were sparse, thickened, and arranged vertically. The most likely diagnosis is ()

A. Eosinophilic granuloma

B. Myeloma

C. Metastatic carcinoma

D. Hemangioma

E. Osteoporosis

81. Single-choice question

The patient is a 45-year-old male. He was admitted to the hospital for half a year because of swelling and pain in his left calf after walking. He felt obvious swelling in his left calf after standing for a long time and walking, and sometimes had pain, which was relieved after rest. If the intraoperative rapid pathology shows a malignant vascular tumor, the best treatment plan is (prompt angiography shows that the tumor is composed of chaotic vascular cavities, with dark staining and no obvious blood vessels. It is considered to be a vascular tumor with a high possibility of malignancy.) ()

A. Simple surgical resection

B. Surgical resection and chemotherapy

C. Radiotherapy

D. Surgical resection and radiotherapy

E. Chemotherapy

82. Single-choice question

The patient is a 50-year-old male. He was diagnosed with mild sacral pain for 1 year and aggravated for 3 days. He had no sensory and motor disorders in both lower limbs and perineum, and often had constipation. The basic examinations required for a clear diagnosis do not include ()

A. Angiography

B. Pelvic X-ray

C. Stool routine

D. Rectal examination

E. CT scan

83. Single-choice question

The patient, male, 66 years old, felt severe pain in the elbow when he reached for something on his right upper limb and was afraid to move. He went to the hospital for emergency X-rays and found a distal humeral fracture with slight displacement. Low-density areas in the bone were visible at both ends of the fracture. He had lost a lot of weight in the past month, had a poor appetite, and often had paroxysmal coughs. The first examination that should be performed next is ()

A. Electrocardiogram

B. Chest X-ray examination

C. Distal humeral CT

D. Distal humeral MRI

E. Ultrasound examination of the liver and gallbladder

84. Single-choice questions

The patient, male, 75 years old, was diagnosed with systemic bone pain. Bone scans revealed multiple bone accumulations throughout the body, and other examinations revealed prostate cancer. Prepare for non-surgical treatment. The local treatment that is effective in suppressing the bone metastasis of prostate cancer is ()

A. Analgesics

B. Chemotherapy

C. Radiotherapy

D. Hormone therapy

E. Bisphosphonates

85. Single-choice questions

The patient is a 75-year-old male who came to the hospital for systemic bone pain. Bone scans revealed multiple bone thickenings throughout the body, and other examinations revealed prostate cancer. He is preparing to take non-surgical treatment. The systemic treatment that is effective in suppressing the bone metastasis of prostate cancer is ()

A. Analgesics

B. Chemotherapy

C. Radiotherapy

D. Hormone therapy

E. Bisphosphonates

86. Single-choice questions

The patient is a 75-year-old male who came to the hospital for systemic bone pain. Bone scans revealed multiple bone thickenings throughout the body, and other examinations revealed prostate cancer. He is preparing to take non-surgical treatment. If bisphosphonates are used for treatment, their main effect is on ()

A. Osteoblasts

B. Osteoclasts

C. Osteoblasts

D. Chondrocytes

E. Fibrocytes

87. Single-choice questions

The patient is a 7-year-old male with intermittent right hip pain for 3 months, accompanied by fever. X-rays and CT scans showed worm-like destruction of the right iliac bone, forming a soft tissue mass. Laboratory tests showed elevated white blood cells. The preliminary diagnosis is Ewing sarcoma. The risk factors for poor prognosis of this tumor do not include ()

A. Tumor located in the pelvis

B. Male patients

C. Tumor diameter > 8cm

D. Tumor volume > 100ml

E. Presence of lung or bone metastasis

88. Single-choice questions

The patient is a 7-year-old male with intermittent right hip pain for 3 months, accompanied by fever. X-rays and CT scans showed worm-like destruction of the right iliac bone, forming a soft tissue mass. Laboratory tests showed elevated white blood cells. The preliminary diagnosis is Ewing sarcoma. For staging, the examination that must be performed in addition to lung CT is ()

A.MRI

B.Bone scan

C.Genetic examination

D.Angiography

E.Biochemical examination

89. Single-choice question

The patient is a 7-year-old male with intermittent right hip pain for 3 months, accompanied by fever. X-rays and CT showed worm-like destruction of the right iliac bone, forming a soft tissue mass. Laboratory tests showed elevated white blood cells. The preliminary diagnosis was Ewing sarcoma. The disease that is most likely to be confused with this diagnosis is ()

A.Osteosarcoma

B.Mesenchymal chondrosarcoma

C.Osteomyelitis

D.Giant cell tumor of bone

E.Enchondroma

90. Single-choice question

The patient is a 23-year-old female. Preoperative pathological biopsy diagnosed osteosarcoma of the upper end of the right tibia and prepared for limb-saving surgery. If you choose allogeneic bone and joint transplantation, it is best to choose ()

A. Heterogeneous allogeneic bone transplantation

B. Homologous fresh allogeneic bone transplantation

C. Homologous frozen or dry frozen allogeneic bone transplantation

D. Fresh bone transplantation from the patient's relatives

E. Fresh bone transplantation from the patient's immediate family members

91. Single choice question

The patient is a 23-year-old female. Preoperative pathological biopsy diagnosed osteosarcoma of the upper right tibia and she was prepared for limb-saving surgery. If better postoperative function is required, () is generally not selected

A. Inactivation and reimplantation

B. Customized artificial joint prosthesis replacement

C. Combined artificial joint prosthesis replacement

D. Allogeneic bone transplantation

E. Joint fusion

92. Single choice question

The patient is a 23-year-old female. Preoperative pathological biopsy diagnosed osteosarcoma of the upper right tibia and she was prepared for limb-saving surgery. Complications after allogeneic bone and joint transplantation do not include ()

A. Rejection infection

B. Allogeneic bone fracture

C. Delayed union and nonunion

D. Joint degeneration

E. Aseptic loosening

93. Single-choice questions

The patient, a 26-year-old female, was diagnosed with right hip discomfort for 2 months. X-rays showed thinning of the cortical bone of the upper part of the right femur, varying degrees of bone expansion, "ground glass" changes in the medullary cavity, and no periosteal reaction. The diagnosis should first consider ()

A. Bone metastasis

B. Chondrosarcoma

C. Bone cyst

D. Bone fibrous dysplasia

E. Fiber dysplasia

94. Single-choice questions

The patient, a 35-year-old female, was diagnosed with general pain for 1 year. Bone scans showed multiple radionuclide concentrations throughout the body, X-rays showed diffuse osteoporosis of the spine, fibrous cysts of the right humeral shaft, intact cortical bone, and no periosteal reaction. Laboratory tests showed elevated blood calcium, and B-ultrasound revealed urinary stones. Further examinations for this patient are ()

A. Lumbar spine MRI

B. Right humerus and pelvis MRI

C. Bone marrow puncture

D. Angiography of right humeral lesions

E. CT scan of the neck

95. Single choice questions

The patient is a 35-year-old female who came to the hospital for treatment due to body pain for 1 year. Bone scan showed multiple radionuclide concentration foci throughout the body, X-ray showed diffuse osteoporosis of the spine, fibrocystic cysts on the right humeral shaft, intact cortical bone, and no periosteal reaction. Laboratory tests showed elevated blood calcium, and B-ultrasound revealed urinary stones. The most likely diagnosis for this is ()

A. Osteoporosis

B. Bone metastasis

C. Multiple giant cell tumors of bone

D. Hyperparathyroidism

E. Multiple myeloma

96. Single choice questions

The patient is a 35-year-old female who was diagnosed with general pain for 1 year. Bone scan showed multiple radionuclide concentrations throughout the body, X-ray showed diffuse osteoporosis of the spine, fibrocystic cysts of the right humeral shaft, intact bone cortex, and no periosteal reaction. Laboratory tests showed elevated blood calcium, and B-mode ultrasound found urinary stones. After the diagnosis, the preferred treatment plan is ()

A. Scrape and bone graft fixation of the right humerus and pelvis

B. Parathyroidectomy

C. Radiotherapy for systemic bone lesions

D. High-dose chemotherapy

E. Anti-osteoporosis treatment

97. Single choice questions

The patient is a 35-year-old female who had swelling and pain in the right knee joint for half a year and could not walk for a long time. Physical examination: swollen knee joint, positive floating patella test, and limited joint movement. X-rays showed no abnormality. The preferred treatment for this patient is ()

A. Plaster fixation

B. Open biopsy

C. Antibiotic treatment

D. Arthroscopic exploration

E. Radiotherapy

98. Single-choice questions

The patient is a 35-year-old female with right knee joint swelling and pain for half a year and cannot walk for a long time. Physical examination: knee joint swelling, positive floating patella test, and limited joint movement. X-rays showed no abnormality. To help with diagnosis, the patient also needs to undergo the following examinations ()

A. CT

B. Joint fluid examination

C. Arthroscopic examination

D. Routine blood test

E. Blood biochemical examination

99. Single-choice questions

The patient is a 38-year-old female with intermittent swelling and pain at the lower end of the right thigh for 2 years. X-rays show osteolytic destruction at the lower end of the right femur, involving the bone end, showing eccentric expansive changes, and no obvious periosteal reaction. The possible prognosis of this tumor is ()

A. Less local recurrence

B. Prone to distant metastasis

C. Slow growth of metastatic tumor

D. Histological characteristics reflect prognosis

E. Poor prognosis

100. Single-choice questions

The patient is a 38-year-old female with intermittent pain in the lower right thigh for 2 years. X-rays showed osteolytic destruction of the lower right femur, involving the bone end, showing eccentric expansive changes, and no obvious periosteal reaction. It seems that the most likely diagnosis is ()

A. Osteosarcoma

B. Ewing sarcoma

C. Chondrosarcoma

D. Giant cell tumor of bone

E. Enchondroma

101. Single-choice question

The patient is a 44-year-old female with a soft tissue tumor in the right thigh, and the pathological diagnosis is liposarcoma. The correct treatment method is ()

A. Surgical resection

B. Chemotherapy

C. Radiotherapy

D. Chemotherapy + radiotherapy

E. Surgical resection, adjuvant radiotherapy and chemotherapy

102. Single-choice question

The patient is a 44-year-old female with a soft tissue tumor in the right thigh, and the pathological diagnosis is liposarcoma. The serious long-term complications of the patient after radiotherapy are ()

A. Wound infection

B. Scar tissue formation

C. Sarcoma formation after radiotherapy

D. Venous reflux disorder

E. Muscle atrophy

103. Single-choice question

The patient is a 44-year-old female with a soft tissue tumor in the right thigh, and the pathological diagnosis is liposarcoma. The following cases do not require radiotherapy ()

A. Tumor marginal resection

B. Tumor diameter> 5cm

C. Tumor recurred multiple times

D. Tumor is close to blood vessels and nerves

E. Extensive resection of low-grade malignant liposarcoma

104, Single-choice questions

The patient is a 55-year-old female. She had a lump on the inner side of her right thigh for more than 20 years. In recent months, she felt that the tumor had increased significantly and was accompanied by chest and rib discomfort. Physical examination: 8cm×10cm lump on the inner side of the right thigh, hard, unable to move, no tenderness. X-ray femur showed no abnormality. Regarding the treatment of this patient, the first choice should be ()

A. Surgery

B. Chemotherapy

C. Radiotherapy

D. Hormone therapy

E. Biological therapy

105, Single-choice questions

The patient is a 55-year-old female. She had a lump on the inner side of her right thigh for more than 20 years. In recent months, she felt that the tumor had increased significantly and was accompanied by chest and rib discomfort. Physical examination: 8cm×10cm lump on the inner side of the right thigh, hard, unable to move, no tenderness. X-ray femur showed no abnormality. To help with the diagnosis, the patient needs to undergo the following tests except ()

A.MRI

B.Lung CT

C.Puncture biopsy

D.ECT

E.Hematological examination

106. Single-choice question

The patient is a 56-year-old female. She was admitted to the hospital for 2 weeks with right hip pain and limping. She got better after rest. Laboratory examination showed normal blood and urine routine. The right hip anteroposterior and lateral X-ray films showed 1.0cm×1.5cm osteolytic changes in the center of the right femoral neck, with no surrounding bone sclerosis. The chest X-ray film found a high-density shadow around the right bronchus with unclear boundaries, about 3cm×6cm. The hip joint MRIT image showed patchy low-signal shadows in the center of the femoral neck, the front of the acetabulum, and the left sacral wing. The inguinal lymph nodes were enlarged. The first biopsy site should be selected ()

A. Bronchoscopy of the right parabronchial tumor biopsy

B. CT-guided femoral neck puncture biopsy

C. Acetabular incision biopsy

D. Sacral puncture biopsy

E. Inguinal lymph node incision biopsy

107, Single-choice questions

The patient was a 56-year-old female who was hospitalized for 2 weeks with right hip pain and limping. She improved after rest. Laboratory tests showed normal blood and urine routine. The right hip anteroposterior and lateral X-ray films showed 1.0cm×1.5cm osteolytic changes in the center of the right femoral neck, with no bone sclerosis around. The most appropriate further examination is ()

A. Electrocardiogram

B. Abdominal ultrasound

C. CT plus 3D reconstruction

D. Bilateral hip ECT examination

E. Bilateral hip MRI

108. Single-choice questions

The patient is a 66-year-old female with right hip pain for 2 months, which is aggravated by activity and night. The diagnosis that must be considered is (suggesting a 4-year history of thyroid cancer. Physical examination: limited right hip movement, positive longitudinal percussion pain in the right lower limb.) ()

A. Osteoporosis

B. Giant cell tumor of bone

C. Osteosarcoma

D. Traumatic fracture

E. Pathological fracture

109. Single-choice questions

The patient is a 66-year-old female with right hip pain for 2 months, which is aggravated by activity and night. The best surgical treatment to relieve symptoms and improve survival is (the patient suddenly experienced severe pain in the right hip when walking, could not stand, and had external rotation and shortening of the right lower limb. X-ray showed a pathological fracture of the right femoral neck.) ()

A. Bone traction

B. Internal fixation treatment

C. Tumor scraping and bone cement filling internal fixation treatment

D. Artificial joint prosthesis replacement

E. Tumor scraping and bone cement type artificial prosthesis replacement

110, single choice questions

Among the clinical manifestations of spinal tuberculosis complicated with paraplegia, the correct one is ()

A. The initial manifestation is a sense of banding in the lesion segment, and then paraplegia occurs

B. Generally, movement disorders appear first

C. Tuberculosis lesions develop slowly, the spinal cord is slowly compressed, and conduction dysfunction gradually occurs. Because the reflex arc is still intact, it manifests as spastic paralysis

D. If the tuberculosis lesions progress quickly, the spinal cord is acutely compressed, and the reflex arc function is lost, it manifests as flaccid paralysis

E. All of the above

111, single choice questions

After the vertebral body of spinal tuberculosis is destroyed, the cold abscess formed will not appear ()

A. Paravertebral abscess

B. Psoas abscess

C. Iliac fossa abscess

D. Deep inguinal abscess

E. Femoral triangle abscess

112, Single choice questions

Spinal tuberculosis is most likely to occur in the spine ()

A. Transverse process

B. Vertebra

C. Spinous process

D. Articular process

E. Lamina

113, Single choice questions

It seems that which of the following is not a sign of stenosing tenosynovitis ()

A. Snapping finger

B. Trigger finger

C. Snapping thumb

D. Drumstick finger

E. Positive fist ulnar deviation test

114, Single choice questions

There are 7 diagnostic criteria for rheumatoid arthritis. How many of them should be present in a typical case ()

A. 3 items

B. 4 items

C. 5 items

D. 6 items

E. 7 items

115, Single choice questions

The main characteristics of benign soft tissue tumors do not include ()

A. Slow growth

B. Clear boundaries and capsule

C. Pain

D. No metastasis

E. Treatment is mainly surgical resection

116. Single-choice questions

The way tumor cells destroy normal tissues does not include ()

A. Loss of blood supply, leading to tissue necrosis, autolysis and phagocytosis

B. Squeezing surrounding normal tissues

C. Enzyme secretion, depolarizing the matrix of connective tissue, causing the collapse of the natural barrier

D. Inflammatory infiltration, weakening surrounding normal tissues

E. Promoting the enhancement of enzyme action, causing connective tissue absorption

117. Single-choice questions

The purpose of surgical treatment of chronic osteomyelitis does not include ()

A. Eliminate sinus tracts

B. Remove large pieces of dead bone

C. Eliminate dead space

D. Close wounds

E. Identify pathogens

118. Single-choice questions

Male, 15 years old, student. Persistent pain in the right knee joint for 3 months. The pain worsens after activity, and the pain is more obvious at night than during the day. Physical examination: The skin temperature of the distal left femur is increased, the veins are distended, and a 4cm×3cm×3cm mass can be touched. It is hard, has unclear boundaries, and has low mobility. X-rays show osteolytic changes in the distal femur, and Codman's triangle can be seen. The most diagnostically valuable examination method for this patient is ()

A. Right femoral MRI

B. Right femoral CT

C. Mass puncture biopsy

D. Right femoral angiography

E. Radionuclide scanning

119, Single-choice questions

Male, 15 years old, student. Persistent pain in the right knee joint for 3 months. The pain worsens after activity, and the pain is more obvious at night than during the day. Physical examination: The skin temperature of the distal left femur is increased, the veins are distended, and a 4cm×3cm×3cm mass can be touched. It is hard, has unclear boundaries, and has low mobility. X-rays show osteolytic changes in the distal femur, and Codman's triangle can be seen. The patient first received high-dose chemotherapy. Which of the following statements is incorrect ()

A. There are more gastrointestinal reactions after chemotherapy

B. Patients should pay attention to preventing infection when their leukocytopenia is less than 3×10/L

C. Adriamycin has no obvious damage to the myocardium

D. Liver function may be impaired after chemotherapy

E. Bone marrow suppression may occur after chemotherapy

120, Single-choice questions

Male, 15 years old, student. Persistent pain in the right knee joint for 3 months. The pain worsens after activity, and the pain is more obvious at night than during the day. Physical examination: The skin temperature of the distal left femur is increased, the veins are distended, and a 4cm×3cm×3cm mass can be touched. It is hard, has unclear boundaries, and has low mobility. X-rays show osteolytic changes in the distal femur, and Codman's triangle can be seen. So the most likely diagnosis for the patient below is ()

A. Giant cell tumor of right femur

B. Osteoblastoma of right femur

C. Osteoid osteoma of right femur

D. Osteosarcoma of right femur

E. Hemangioma of right femur

121. Single-choice question

Male, 23 years old, persistent dull pain in the lower right thigh for more than 1 month. Physical examination: swelling and tenderness on the inner side of the lower right thigh. X-ray showed a bone destruction area on the inner side of the lower right femur, which grew in a soap bubble-like expansion, with localized, rounded lesions and thinning of the bone cortex. If it is finally diagnosed as osteosarcoma, the best treatment plan is ()

A. Chemotherapy alone

B. Radiotherapy alone

C. Amputation alone

D. Tumor resection and artificial joint replacement

E. Chemotherapy-tumor resection-chemotherapy

122. Single-choice question

Male, 23 years old, persistent dull pain in the lower right thigh for more than 1 month. Physical examination: swelling and tenderness on the inner side of the lower right thigh. X-ray showed a bone destruction area on the inner side of the lower right femur, which grew in a soap bubble-like expansion, with localized, rounded lesions and thinning of the bone cortex. So the best treatment measure is ()

A. Chemotherapy alone

B. Amputation alone

C. Scraping and bone grafting

D. Radiotherapy alone

E. Anti-inflammatory and anti-tuberculosis

123. Single-choice question

Male, 23 years old, persistent dull pain in the lower right thigh for more than 1 month. Physical examination: swelling and tenderness on the inner side of the lower right thigh. X-ray showed a bone destruction area on the inner side of the lower right femur, which grew in a soap bubble-like expansion pattern. The lesion was localized, round, and the bone cortex was thinned. So the disease that should be considered first is ()

A. Bone tuberculosis

B. Osteomyelitis

C. Osteosarcoma

D. Osteochondroma

E. Giant cell tumor of bone

124. Single-choice question

Male, 10 years old. Pain and swelling in the lower right thigh, increased skin temperature, accompanied by high fever of 39.5℃. Clinically suspected to be acute suppurative osteomyelitis. If the diagnosis is clear, the most critical treatment method is ()

A. Bed rest

B. Symptomatic treatment

C. Limb immobilization

D. Intravenous infusion, nutritional support

E. Application of sensitive antibiotics + drilling drainage

125, Single choice questions

Male, 10 years old. Pain and swelling at the lower end of the right thigh, increased skin temperature, accompanied by high fever of 39.5℃. Clinically suspected to be acute suppurative osteomyelitis. In the physical examination of the child, the most clinically significant sign is ()

A. Increased skin temperature at the lower end of the right femur

B. Deep tenderness at the lower end of the right femur (metaphysis)

C. Swelling at the lower end of the right femur

D. Local vascular filling and engorgement

E. Limited extension and flexion of the right knee joint

126, Single choice questions

Male, 11 years old. Pain after left lower limb trauma for 10 days, aggravated with limited activity and fever for 3 days, admitted to the hospital. It was due to the injury of the left thigh while running 10 days ago, obvious pain, limited activity, and slightly relieved after bed rest. The pain worsened 3 days ago, accompanied by fever, with a temperature as high as 40℃. He was treated with intravenous penicillin in a local hospital. X-ray examination showed no abnormalities. Physical examination: T38.8℃, P110 times/min. The circumference of the left thigh is 35cm, the circumference of the right thigh is 32cm, and the circumference of both calves is 25cm. The dorsalis pedis artery pulsates well and the skin feels normal. At this time, the inappropriate treatment measures are (Hint: No pus was drawn from the local puncture, only a small amount of non-coagulated blood was drawn, and no bacteria were found in Gram staining. The patient had obvious chills, high fever, and a body temperature of 40.0℃. Blood was drawn immediately for blood culture.) ()

A. Combined use of large doses of broad-spectrum antibiotics

B. Correction of water and electrolyte metabolism disorders, small and multiple blood transfusions

C. Wait for the results of bacterial culture to determine the next step of treatment

D. Local plaster fixation

E. Physical cooling

F. Systemic supportive treatment

127, Single choice questions

Male, 11 years old. The patient had pain in the left lower limb after trauma for 10 days, which worsened with limited activity and fever for 3 days before being admitted to the hospital. The patient was injured in the left thigh while running 10 days ago, with obvious pain and limited activity, which was slightly relieved after bed rest. The pain worsened 3 days ago, accompanied by fever, with a body temperature as high as 40℃. He was treated with intravenous penicillin in a local hospital. No abnormalities were found in the X-ray examination. Physical examination: T38.8℃, P110 times/min. The circumference of the left thigh is 35cm, the circumference of the right thigh is 32cm, and the circumference of both calves is 25cm. The dorsalis pedis artery pulsates well and the skin sensation is normal. According to the examination results, the most critical treatment measures should be taken (Hint: MRI examination showed multiple patchy long T1 and long T2 signals in the medullary cavity of the middle and lower part of the left femur, swelling of the surrounding soft tissue, blurred fat gaps, and a small amount of effusion in the joint cavity.) ()

A. Repeated puncture and pus extraction

B. Timely adjustment of antibiotics and dosage

C. Prevent pathological fractures

D. Local bone cortical window drainage and continuous irrigation

E. Prevent and treat fluid metabolism disorders

F. Physical cooling

128, Single choice questions

Male, 11 years old. Pain after left lower limb trauma for 10 days, aggravated with limited activity and fever for 3 days, admitted to the hospital. It was caused by a left thigh injury while running 10 days ago, obvious pain, limited activity, and slightly relieved after bed rest. The pain worsened 3 days ago, accompanied by fever, and the body temperature was as high as 40℃. He was treated with intravenous penicillin in a local hospital. X-ray examination did not find any abnormalities. Physical examination: T38.8℃, P110 times/min. The circumference of the left thigh is 35cm, the circumference of the right thigh is 32cm, and the circumference of both calves is 25cm. The dorsalis pedis artery pulse is good and the skin sensation is normal. At present, the preliminary clinical diagnosis is suppurative osteomyelitis of the lower femur. The main basis for early diagnosis is (prompt: examination results: WBC22.44×10/L, N87%, RBC3.75×10/L, Hb90.6/L, PLT386×10/L, C-reactive protein182mg/L, ESR110mm/h, serum potassium3.4mmol/L, serum sodium130.2mmol／L.) ()

A. Metaphyseal pain and deep tenderness

B. X-ray shows worm-like bone destruction and periosteal hyperplasia

C. Systemic poisoning symptoms, high fever

D. Limb swelling and pain

E. Local layered puncture draws pus under the periosteum or in the bone marrow cavity

F. ESR, C-reactive protein, and white blood cell counts are elevated

129, Single choice questions

Male, 11 years old. Pain after left lower limb trauma for 10 days, aggravated with limited activity and fever for 3 days, admitted to hospital. The patient was injured in the left thigh while running 10 days ago. He had obvious pain and limited mobility. The pain was slightly relieved after bed rest. The pain worsened 3 days ago, accompanied by fever, with a temperature as high as 40℃. He was treated with intravenous penicillin in a local hospital. X-ray examination showed no abnormalities. Physical examination: T38.8℃, P110 times/min. The circumference of the left thigh is 35cm, the circumference of the right thigh is 32cm, and the circumference of both calves is 25cm. The dorsalis pedis artery pulsates well and the skin feels normal. The most likely pathogen in bacterial culture is (Hint: The collected pus is sent for bacterial culture + drug sensitivity test.) ()

A. Staphylococcus aureus

B. Streptococcus group B

C. Pneumococcus

D. Escherichia coli

E. Staphylococcus aureus

F. Streptococcus

G. Proteus

130, Single choice questions

Male, 15 years old. He has proximal tibia pain and swelling for 1 month, obvious pain at night, and no obvious history of trauma. The patient developed local swelling and pain and abnormal activity after falling. The treatment methods that can be used are ()

A. Bed rest

B. Open surgery

C. Puncture biopsy

D. Radiotherapy

E. Chemotherapy

F. Physical therapy

131. Single choice questions

Male, 15 years old. There was pain and swelling in the proximal tibia for 1 month, obvious pain at night, and no obvious history of trauma. The X-ray film taken at the consultation showed: a cystic translucent area in the proximal left tibia with clear boundaries. The most likely diagnosis is ()

A. Osteochondroma

B. Osteosarcoma

C. Myositis ossificans

D. Bone tuberculosis

E. Chronic osteomyelitis

F. Giant cell tumor of bone

132. Single choice questions

Male, 24 years old, had a progressively enlarged hard swelling and pain in the right wrist for 3 months. The pain symptoms were significantly aggravated after minor trauma. X-ray film shows: There is an eccentric osteolytic bone destruction area at the distal end of the radius, accompanied by pathological fractures, cortical bone expansion, and "soap bubble-like changes". However, the most likely diagnosis is ()

A. Osteosarcoma

B. Osteoid osteoma

C. Enchondroma

D. Synovial sarcoma

E. Giant cell tumor of bone

133, Single choice question

Male, 24 years old, has a progressively enlarged hard swelling and pain in the right wrist for 3 months. The pain symptoms are significantly aggravated after minor trauma. X-ray film shows: There is an eccentric osteolytic bone destruction area at the distal end of the radius, accompanied by pathological fractures, cortical bone expansion, and "soap bubble-like changes". So the main tumor cells in this tumor tissue are ()

A. Giant cells (osteoclasts)

B. Type I stromal cells

C. Chondrocytes

D. Synovial cells

E. Osteoblasts

134, Single choice question

Male, 25 years old. Likes tennis. Pain in the lateral side of the right elbow joint for 1 month, aggravated for 3 days. Weakness in holding objects, pain in twisting towels. In addition to localized tenderness on the lateral side of the right elbow joint, which of the following tests is most helpful for diagnosis ()

A. Froment test

B. Mills test

C. Finkelstein test

D. Gaenslen test

E. Dugas test

135, Single choice questions

Male, 28 years old. Pain in the right hip, accompanied by low-grade fever in the afternoon, night sweats, lack of appetite and weight loss for 1 year. Physical examination: The right hip joint is flexed and deformed, with no obvious swelling in appearance, limited flexion and extension, and Thomas sign (+). ESR56mm/h. X-ray examination: The right hip joint space is narrowed, the joint surface has worm-like bone destruction, and the right acetabulum has a 2.5cm cavity with dead bone formation. During hospitalization, the patient's body temperature was 39℃, and an 8cm×6cm mass appeared on the greater trochanter of the right hip, with red and hot skin on the surface and a sense of fluctuation. To understand the nature of the mass, the correct choice of the following puncture site is ()

A. The place where the mass fluctuates

B. The lower part of the mass

C. The higher part of the mass

D. The healthy skin around the mass

E. As long as the pus can be extracted, the needle insertion site is not limited

136, Single choice questions

Male, 28 years old. Right hip pain, accompanied by low-grade fever in the afternoon, night sweats, lack of appetite and weight loss for 1 year. Physical examination: The right hip joint is flexed and deformed, with no obvious swelling in appearance, limited flexion and extension, and Thomas sign (+). ESR56mm/h. X-ray examination: The right hip joint space is narrowed, the joint surface has worm-like bone destruction, and the right acetabulum has a 2.5cm cavity with dead bone formation. During the hospitalization, the patient's body temperature was 39℃, and an 8cm×6cm mass appeared in the greater trochanter of the right hip, with red and hot skin on the surface and a sense of fluctuation. To understand After 4 weeks of anti-tuberculosis treatment, the patient's spirit and appetite improved, ESR20mm/h. At this time, the treatment should be ()

A. Joint replacement surgery

B. Hip "R" shaped plaster fixation

C. Continuous skin traction of the affected limb

D. Lesion removal

E. Joint puncture and pus extraction, injection of anti-tuberculosis drugs

137, Single choice questions

Male, 28 years old. Right hip pain, accompanied by low-grade fever in the afternoon, night sweats, lack of appetite and weight loss for 1 year. Physical examination: The right hip joint is flexed and deformed, with no obvious swelling in appearance, limited flexion and extension, Thomas sign (+). ESR56mm/h. X-ray examination: The right hip joint space is narrowed, the joint surface has worm-like bone destruction, and the right acetabulum has a 2.5cm cavity with dead bone formation. During the hospitalization, the patient's body temperature was 39℃, and an 8cm×6cm mass appeared on the greater trochanter of the right hip, with red and hot skin on the surface and a sense of fluctuation. To understand the following options, the most likely diagnosis is ()

A. Suppurative hip arthritis

B. Hip synovial tuberculosis

C. Hip bone tuberculosis

D. Total hip tuberculosis

E. Rheumatoid hip arthritis

138, Single choice questions

Male, 53 years old, wheelchair pushed into the outpatient clinic, complained of numbness of the right thumb and index finger, weakness of the lower limbs, and unsteady walking for 1 year without obvious inducement, which worsened for 3 weeks. Physical examination revealed increased muscle tension in the lower limbs and positive pyramidal tract signs. However, the most reasonable treatment method at present is ()

A. Bed rest, reduce neck activity

B. Surgery as soon as possible

C. Medication, physical therapy, traction and other conservative treatments

D. Strengthen neck muscle exercise and enhance cervical spine stability

E. Wear a neck collar and reexamine after 1 month

139, Single choice questions

Male, 53 years old, wheelchair pushed into the outpatient clinic, complained of numbness of the right thumb and index finger, weakness of the lower limbs, and unsteady walking for 1 year without obvious inducement, which worsened for 3 weeks. Physical examination revealed increased muscle tone in the lower limbs and positive pyramidal tract signs. The following possible diagnoses include ()

A. Radiculopathy

B. Vertebral artery

C. Myelopathy

D. Thoracic outlet syndrome

E. Thoracic tumor

140, Single choice questions

Male, 5 years old. Sudden high fever and chills for 3 days. Physical examination: temperature 39.1℃, pulse 118 beats/min. The left knee joint was obviously red, swollen and tender, with severe pain during movement, and floating patella test (+). The current treatment measures that should not be taken are ()

A. Local immobilization

B. Application of high-dose antibiotics

C. Intra-articular injection of antibiotics

D. Systemic supportive treatment

E. Incision and drainage

141. Single-choice question

Male, 7 years old. Sudden severe pain in the lower right thigh accompanied by chills, high fever, and restlessness for 3 days. Physical examination: body temperature 39.8℃. Tenderness in the distal right thigh, refusal to press, and semi-flexed knee joint. The most common pathogen of this disease is ()

A. Group B Streptococcus

B. Staphylococcus aureus

C. Escherichia coli

D. Streptococcus

E. No bacterial growth

142. Single-choice question

Male, 7 years old. Sudden severe pain in the lower right thigh accompanied by chills, high fever, and restlessness for 3 days. Physical examination: body temperature 39.8℃. Tenderness in the distal right thigh, refusal to press, and semi-flexed knee joint. The main basis for early diagnosis is ()

A. high fever, limb swelling and pain

B. X-ray film

C. CT examination

D. Increased total white blood cell count and neutrophil count

E. Local layered puncture to extract pus

143, single choice questions

Male, 7 years old. Sudden severe pain under the right thigh with chills, high fever, irritability for 3 days. Physical examination: body temperature 39.8℃. Distal tenderness of the right thigh, refusal to press, knee joint is semi-flexed. The most likely diagnosis of the following options is ()

A. Rheumatic knee arthritis

B. Suppurative knee arthritis

C. Suppurative osteomyelitis of the lower end of the femur

D. Traumatic knee arthritis

E. Tuberculous knee arthritis

144, single choice questions

Male, 8 years old. Pain and swelling in the lower part of the left thigh for 1 week. Physical examination: body temperature 39.5℃, high local skin temperature, deep tenderness, ineffective anti-infection treatment, local puncture to extract a small amount of purulent fluid. According to the child's condition, the treatment measures that should not be taken are ()

A. Combined use of high-dose antibiotics

B. Local immobilization

C. Systemic supportive therapy

D. Local decompression and drainage

E. Lesion removal

145. Single-choice questions

Female, 16 years old, has a lump below the right knee, which has gradually increased in size over the past 5 years, painless, and normal gait. X-rays revealed a lump on the upper inner side of the right tibia, with trabeculae at the base connected to the tibia, the density of the base-top cover decreased, and the border was still recognizable. Based on the above symptoms, the most likely diagnosis is ()

A. Osteosarcoma

B. Giant cell tumor of bone

C. Osteoma

D. Osteochondroma

E. Bone metastasis

146. Single-choice questions

Female, 16 years old, has a lump below the right knee, which has gradually increased in size over the past 5 years, painless, and normal gait. X-rays revealed a lump on the upper inner side of the right tibia, with trabeculae at the base connected to the tibia, the density of the base-top cover decreased, and the border was still recognizable. However, if the tumor suddenly grows faster, and the X-ray shows a vague cotton-like calcification shadow at the edge of the tumor, the treatment should be ()

A. Observation

B. Tumor resection

C. Local extensive resection or amputation

D. Radiotherapy

E. Chemotherapy

147, Single-choice question

Female, 24 years old. Recently, I found pain in the left knee joint, difficulty walking, which was relieved by rest. I felt that there was a lump and tenderness on the inner side of the left calf. Physical examination: The movement of the left knee joint is slightly limited, and the inner side of the upper tibia is swollen and tender. X-ray shows a soap bubble-like shadow on the inner side of the upper end of the left tibia, which grows in an expansive manner. The appropriate treatment method for this case is ()

A. Rest and nutrition

B. Broad-spectrum antibiotic treatment

C. Surgical treatment

D. Anti-tuberculosis treatment

E. Physical therapy

148, Single-choice question

Female, 24 years old. Recently, I found pain in the left knee joint, difficulty walking, which was relieved by rest. I felt that there was a lump and tenderness on the inner side of the left calf. Physical examination: The movement of the left knee joint is slightly limited, and the medial side of the upper tibia is swollen and tender. X-ray shows a soap bubble-like shadow on the medial side of the upper left tibia, which grows in an expansive manner. So the most likely diagnosis in this case is ()

A. Bone tuberculosis

B. Osteomyelitis

C. Osteonecrosis

D. Giant cell tumor of bone

E. Osteochondroma

149, Single choice questions

Female, 33 years old. In the past month, back pain has gradually appeared, accompanied by low fever and night sweats. There is a history of pulmonary tuberculosis. Physical examination: There is obvious tenderness in the 11th and 12th thoracic spinous processes. The first examination to be performed is ()

A. Blood routine and erythrocyte sedimentation rate

B. Thoracic and lumbar X-ray

C. CT

D. OT test

E. Radionuclide bone scan

150, Single choice questions

Female, 33 years old. In the past month, back pain has gradually appeared, accompanied by low fever and night sweats. There is a history of pulmonary tuberculosis. Physical examination: obvious tenderness of thoracic spinous processes 11-12. After the diagnosis is confirmed, the treatment measures that should not be taken at present are ()

A. Regular anti-tuberculosis treatment, pay attention to check whether there are tuberculosis lesions in other parts of the body

B. Rest on a hard bed

C. Immediate surgery, lesion removal

D. Systemic supportive treatment

E. Symptomatic treatment

151. Single-choice questions

Female, 33 years old. In the past month, back pain has gradually appeared, accompanied by low fever and night sweats. There is a history of pulmonary tuberculosis. Physical examination: obvious tenderness of thoracic spinous processes 11-12. The most helpful physical examination test is ()

A. Dugas sign

B. Straight leg raising test

C. Trendelenburg sign

D. Pick-up test

E. Thomas sign

152. Single-choice questions

Female, 50 years old. Right shoulder pain, limited lifting and abduction of the right upper limb for 8 months. Physical examination: no redness, swelling, or increased skin temperature around the shoulder, and pain radiates to the neck, ears, forearms, and hands. The most likely diagnosis is ()

A. Osteoarthritis of shoulder joint

B. Periarthritis of shoulder

C. Tuberculosis of shoulder joint

D. Cervical spondylosis

E. Rheumatoid arthritis

153, Single choice question

Which of the following signs is positive for radial styloid stenosing tenosynovitis ()

A. Finkelstein test

B. Mills test

C. Lasegue test

D. Thomas test

E. Dugas test

154, Single choice question

The chemotherapy drug that is prone to cause adverse cardiac reactions is ()

A. Adriamycin

B. Cisplatin

C. Methotrexate

D. Ifosfamide

E. Bleomycin

155, Single choice question

The treatment method for chondrosarcoma is ()

A. Radiotherapy

B. Chemotherapy

C. Neoadjuvant chemotherapy

D. Complete tumor resection

E. Immunotherapy

156, Single choice question

The most important prognostic factor for soft tissue sarcoma is ()

A. Tumor size

B. Tumor location

C. Histological grade

D. Histological type of tumor

E. Clinical course

157, Single choice questions

The diagnosis of soft tissue tumors is based on ()

A. Medical history

B. Clinical manifestations

C. Imaging manifestations

D. Intraoperative naked eye findings

E. Pathological diagnosis

158, Single choice questions

The representative drugs of the three steps in the three-step cancer pain treatment plan are ()

A. Aspirin, codeine and morphine

B. Paracetamol, codeine and morphine

C. Paracetamol, codeine and morphine

D. Aspirin, codeine and morphine

E. Tramadol, MSContin, OxyContin

159, Single choice questions

Except for the common sites of pigmented villonodular synovitis ()

A. Knee joint

B. Hip joint

C. Ankle joint

D. Wrist joint

E. Sternoclavicular joint

160, Single choice questions

The so-called bone cysts are prone to ()

A. Long tubular bone shaft

B. Long tubular bone metaphysis

C. Long tubular bone end

D. Short tubular bones of hands and feet

E. Axial bones

161. Single-choice question

The chemotherapy necrosis rate of osteosarcoma, which indicates a better prognosis of the tumor, is ()

A. <10%

B. >40%

C. <40%

D. >60%

E. >90%

162. Single-choice question

The X-ray features of advanced hip tuberculosis are ()

A. Disappearance of femoral head

B. Pathological posterior dislocation

C. Progressive narrowing of joint space

D. Marginal bone destruction

E. Swollen joint capsule

163. Single-choice question

The treatment method with better effect for stenosing tenosynovitis is ()

A. Physical therapy

B. Restriction of activities and plaster fixation

C. Physical therapy plus oral medication

D. Local application of wound-wetting and pain-relieving ointment

E. Local blockade of prednisone acetate

164. Single-choice question

The correct clinical features of marginal vertebral tuberculosis are ()

A. More common in adults

B. Common in the lumbar spine

C. Lesions are limited to the upper and lower edges of the vertebral body, but quickly invade the intervertebral disc and adjacent vertebral bodies

D. Intervertebral disc destruction is a characteristic of this disease, which narrows the intervertebral space

E. All of the above

165, Single-choice questions

The following is not an indication for surgery for bone and joint tuberculosis ()

A. Obvious dead bone and abscess formation

B. Sinus pus that does not heal for a long time

C. Early full-joint tuberculosis, in order to save the joint

D. Spinal tuberculosis causes spinal cord compression

E. Severe systemic poisoning symptoms, weakness, severe anemia

166, Single-choice questions

The wrong clinical manifestations of bone and joint tuberculosis are ()

A. Low fever, weight loss, night sweats, anemia

B. Local swelling and pain

C. Increased erythrocyte sedimentation rate

D. Multiple joint lesions

E. Cold abscesses

167, Single-choice questions

Among the following diagnostic points for bone and joint tuberculosis, the incorrect one is ()

A. History of tuberculosis

B. Spinal pain, stiffness, deformity, compression signs, positive pick-up test

C. Pain, swelling, lameness, and limited movement in the joints

D. Although there is anemia, increased ESR, and positive OT test, there may be no symptoms of tuberculosis poisoning

E. X-ray examination shows narrowing of the joint space, bone destruction and other lesions

168, Single-choice questions

The following statement about soft tissue sarcoma is incorrect ()

A. Extraskeletal connective tissue originating from the mesoderm

B. Neural tissue sarcoma originating from the neuroectoderm has similar clinical and pathological characteristics to soft tissue sarcoma and is also classified as soft tissue sarcoma

C. The imaging manifestations of soft tissue sarcoma are mostly non-specific

D. MRI examination can determine the tissue type of soft tissue sarcoma

E. In soft tissue sarcoma, both the grading of the lesion and the determination of the origin of the tissue are important

169, Single-choice questions

Which of the following treatments for acute suppurative arthritis is incorrect ()

A. Combined use of sufficient effective antibiotics

B. Local fixation and supportive treatment

C. Daily superficial joint puncture, drainage, flushing and injection

D. Deep joints are best incised and drained, closed irrigation therapy

E. Acute pathological dislocation is immediately incised and reduced

170, Single choice questions

Which of the following X-ray features of spinal tuberculosis forming cold abscesses is incorrect ()

A. Tracheal displacement is shown on the lateral view of the cervical spine

B. The lateral view of the cervical spine shows widening of the prevertebral soft tissue shadow

C. The thoracic spine AP view shows widening of the paravertebral soft tissue shadow

D. The lumbar AP view shows blurred shadow of the psoas major muscle

E. The lumbar AP view shows widening of the psoas major muscle shadow

171, Single choice questions

The following diagnostic basis for chronic osteomyelitis is incorrect ()

A. Recurrent attacks

B. The affected limb is shorter than the healthy limb

C. There are long-term non-healing sinuses

D. Dead bone is discharged from the sinuses

E. The skin of the affected limb is thin and dark in color

172. Single-choice questions

The following clinical characteristics of ankylosing spondylitis (AS) are incorrect ()

A. Common in young people, more common in men, and the incidence increases with age

B. The HLA-B27 positive rate is as high as 90% to 96%

C. Mainly invades the sacroiliac joint and spine

D. There are obvious low back pain, and the serum rheumatoid factor is negative

E. X-ray manifestations of spinal lesions: The early vertebrae may be "square" vertebrae, and the lesions develop to form bone bridges between vertebrae, presenting the most characteristic "bamboo-like" spine

173. Single-choice questions

The following clinical significance of knee joint diseases and related examinations is incorrect ()

A. Knee joint effusion: floating patella test (+)

B. Posterior cruciate ligament rupture: anterior drawer test (+)

C. Posterior cruciate ligament rupture: posterior drawer test (+)

D. Meniscus injury: McMurray test (+)

E. Chondromalacia patella: patellar friction test (+)

174, Single choice questions

The following X-ray manifestations of knee tuberculosis are incorrect ()

A. Swelling of suprapatellar bursa and soft tissue

B. Narrowing or disappearance of joint space

C. Anterior tibial dislocation

D. Blurred bone with frosted glass-like appearance

E. Erosion and destruction of marginal bone

175, Single choice questions

Which of the following drugs is not a narcotic drug ()

A. Methadone

B. MSContin

C. Buprenorphine

D. Aidone

E. OxyContin

176, Single-choice question

Which of the following imaging examinations has a low diagnosis rate for bone and joint tuberculosis ()

A. X-ray examination

B. Radionuclide bone scan

C. CT examination

D. Ultrasound examination

E. MRI examination

177, Single-choice question

Which of the following tumors is not sensitive to radiotherapy ()

A. Bone hemangiopericytoma

B. Bone hemangioendothelioma

C. Angiosarcoma

D. Malignant schwannoma

E. Myeloma

178, Single-choice question

The thoracic spine X-ray shows that the vertebral body is evenly flattened into sheets. Should be suspected ()

A. Spinal tuberculosis

B. Ankylosing spondylitis

C. Intervertebral disc herniation

D. Spinal stenosis

E. Eosinophilic granuloma

179, Single choice question

Common treatments for desmoid tumors include ()

A. Surgery

B. Surgery + radiotherapy

C. Radiotherapy + chemotherapy

D. Surgery + chemotherapy

E. Radiotherapy

180, Single choice question

The most common age group for Ewing sarcoma is ()

A. 5 to 20 years old

B. 20 to 30 years old

C. 30 to 40 years old

D. 40 to 50 years old

E. Over 50 years old

181. Single-choice question

Which of the following is incorrect about the pathological changes of bone and joint tuberculosis? ()

A. Initially, it is simple synovial tuberculosis or simple bone tuberculosis, the latter is more common

B. Initially, the joint cartilage surface is intact

C. Tuberculosis lesions can further develop and break into the joint cavity

D. Joint tuberculosis can break and form sinus tracts

E. No sequelae can be left after cure

182. Single-choice question

Which of the following is incorrect about synovial sarcoma? ()

A. Slow growth

B. Rare, low incidence

C. Pain is the main symptom

D. Irregular calcification may occur in the tumor

E. Treatment is mainly surgical resection

183. Single-choice question

Which of the following is incorrect about the clinical manifestations of knee tuberculosis? ()

A. Low fever

B. Increased erythrocyte sedimentation rate

C. Positive floating patella test

D. Positive object picking test

E. Knee flexion contracture

184. Single-choice question

The survival period of patients undergoing internal fixation stabilization surgery to prevent pathological fractures of proximal femoral bone metastases should not be less than ()

A. 1 month

B. 2 months

C. 3 months

D. 6 months

E. 12 months

185, Single-choice questions

The most common site of primary bone tumors ()

A. Vertebral body

B. Proximal humerus

C. Bone end near knee joint

D. Pelvis

E. Proximal femur

186, Single-choice questions

In the Enneking staging system, T stands for ()

A. Tumor size

B. Anatomical site of the tumor

C. Tumor metastasis

D. Histological grade

E. Tumor boundary

187, Single-choice questions

In the laboratory examination of bone tumors, the wrong one is ()

A. Extensive osteolytic metastasis or bone destruction, serum calcium is often elevated

B. Osteogenic bone tumor, serum alkaline phosphatase is elevated

C. Prostate cancer spread, serum acid phosphatase is elevated

D. Plasma cell myeloma, total protein concentration is elevated

E. Serum alkaline phosphatase is normal, malignant bone tumors can be excluded

188, single choice questions

The most common benign bone tumor below is ()

A. Myeloma

B. Osteochondroma

C. Giant cell tumor of bone

D. Bone cyst

E. Osteoma

189, single choice questions

The most common malignant bone tumor below is ()

A. Osteosarcoma

B. Chondrosarcoma

C. Fibrosarcoma

D. Ewing sarcoma

E. Chordoma

190, single choice questions

The X-ray manifestations of early hip tuberculosis generally do not show ()

A. Localized osteoporosis

B. Pathological dislocation

C. Progressive narrowing of the joint space

D. Marginal bone destruction

E. Swollen joint capsule

191. Single-choice questions

The chemotherapy drug that is cell cycle-specific is ()

A. Adriamycin

B. Cisplatin

C. Ifosfamide

D. MTX

E. Nitrogen mustard

192. Multiple-choice questions

The patient is a 17-year-old male with pain, swelling and joint movement disorder below the left knee for 2 months. Physical examination: The limb below the left knee joint is 3cm thicker than the contralateral side, with high surface skin temperature, venous distension and local tenderness. X-rays show osteolysis and osteoblastic destruction of the left tibial metaphysis, with solar radiation-like periosteal reaction and soft tissue masses. Alkaline phosphatase 485U/L.

The main treatment methods for this patient include (prompt that the patient's pathological biopsy showed immature osteoid matrix and spindle-shaped cells.) ()

A. Surgery

B. Radiotherapy

C. Chemotherapy

D. Targeted therapy

E. Biological therapy

193, multiple choice questions

The patient is a 17-year-old male with pain, swelling and joint movement disorder below the left knee joint for 2 months. Physical examination: The limb below the left knee joint is 3cm thicker than the contralateral side, the surface skin temperature is high, and venous distension and local tenderness can be seen. X-rays show osteolysis and osteoblastic destruction of the left tibial metaphysis, and solar radiation-like periosteal reaction and soft tissue masses can be seen. Alkaline phosphatase 485U/L.

If the patient needs chemotherapy, the drugs available include ()

A. Adriamycin

B. Cisplatin

C. Methotrexate

D. IFO

E. CTX

194, multiple choice questions

The patient is a 42-year-old male. The main cause is dull pain and discomfort in the lower end of the right thigh for 2 years, which gradually worsens and swells. There is a history of right knee joint trauma in the past.

The surgical treatment options for the disease are ()

A. Intracapsular resection

B. Intracapsular resection plus adjuvant therapy

C. Marginal resection

D. Marginal resection plus adjuvant therapy

E. Wide resection

195. Multiple choice questions

The patient is a 42-year-old male. The main cause is dull pain and discomfort in the lower end of the right thigh for 2 years, which gradually worsens and swells. There is a history of right knee joint trauma in the past.

The examinations required to confirm the diagnosis and determine the treatment plan should include (prompt physical examination: the lower end of the right thigh is 2cm thicker than the opposite side. X-ray shows eccentric osteolytic destruction of the lower end of the right femur, thinning and expansion of the cortex, soap bubble-like changes, no periosteal reaction and soft tissue mass shadow.) ()

A. Blood routine

B. B-type ultrasound

C. CT and MRI

D. Bone scan

E. Angiography

196, multiple choice questions

The patient is a 14-year-old female. She had swelling and pain in the lower part of her left thigh after a bruise for 1 month. The symptoms have worsened in the past 2 weeks, especially at night, and her body temperature is normal.

In order to clarify the diagnosis and surgical staging, the following examinations should be performed (X-ray films show uneven density, unclear boundaries, cortical bone destruction, soft tissue masses, and inconspicuous Codman triangles at the lower femoral metaphysis.) ()

A. Routine blood test

B. Biopsy of the lesion

C. Immediate biochemical examination

D. MRI of the middle and lower segments of the left femur

E. Ultrasound examination of the middle and lower segments of the left femur

197. Multiple choice questions

The patient is a 48-year-old female. She was admitted to the hospital for 1 month with pain in the front of the left thigh. The pain is dull, not relieved by rest, and not significantly aggravated by activity. She had a history of thyroid tumor resection 2 years ago, and the pathological results of the resected specimen showed papillary thyroid cancer, which was well differentiated.

Further laboratory tests should include (prompt physical examination: no mass was palpated in the neck examination, and a scar after thyroidectomy was visible in the front of the neck, about 10 cm long. Both lower limbs were well mobile, with normal muscle strength and no sensory impairment. Physiological reflexes of the lower limbs were present, and pathological reflexes were not elicited. Mild tenderness in the middle and upper part of the left thigh, obvious percussion pain, low skin temperature, and no venous dilatation on the surface. Imaging manifestations: X-ray examination showed that the bone density in the range of 3 cm within the medullary 2 cm below the trochanter of the middle and upper part of the left femur was reduced, the bone cortex was slightly thinned, and the boundary of the low-density area was unclear.) ()

A. Blood routine

B. Urinalysis

C. Thyroid function

D. Chest X-ray

E. Thyroid ultrasound

198. Multiple choice questions

The patient is a 48-year-old female. She was admitted to the hospital for 1 month of pain in the front of the left thigh. The pain was dull, not relieved by rest, and not significantly aggravated after activity. There was a history of thyroid tumor resection 2 years ago. The pathological results of the resected specimen showed papillary thyroid cancer, which was well differentiated.

Further treatment strategies include (the biopsy report indicates synovial sarcoma with moderate differentiation. The patient insisted on limb preservation.) ()

A. Preoperative chemotherapy can be performed first, followed by local extended resection

B. Tumor reduction surgery such as partial resection or intratumoral curettage first, followed by local radiotherapy

C. Extended resection, and whether to perform radiotherapy or chemotherapy is determined based on postoperative pathology

D. Radionuclide therapy first, and then surgery or radiotherapy or chemotherapy is determined based on efficacy

E. Radiotherapy is performed to shrink the tumor first, followed by surgical resection

F. Chemotherapy combined with radiotherapy

199. Multiple choice questions

The patient is a 48-year-old female. She was hospitalized for 1 month with pain in the front of the left thigh. The pain is dull, not relieved by rest, and not significantly aggravated by activity. She had a history of thyroid tumor resection 2 years ago, and the pathological results of the resected specimen showed papillary thyroid cancer, which was well differentiated.

The diseases that can be excluded are (X-ray shows no bone abnormalities, MRI shows T-weighted medium signal, T-weighted high signal, uneven signal, and high signal when fat is suppressed.) ()

A. Desmoid tumor

B. Cyst

C. Synovial tumor

D. Fat-derived tumor

E. Xanthomas

200, multiple choice questions

The patient is a 48-year-old female. She was admitted to the hospital for 1 month with pain in the front of the left thigh. The pain is dull, not relieved by rest, and not significantly aggravated after activity. There was a history of thyroid tumor resection 2 years ago, and the pathological results of the resected specimens showed papillary thyroid cancer, which was well differentiated.

The treatment measures that can be selected are (prompt that the pathological results of bone puncture biopsy revealed thyroid duct-like structures, cells with obvious developmental abnormalities, large nuclei, and darkly stained cells are easy to see. Diagnosis: ① postoperative thyroid cancer; ② single left proximal femoral bone metastasis.) ()

A. Continue regular observation and follow-up

B. Tumor segment resection + intramedullary nail internal fixation + bone cement filling of bone defects

C. Lesion scraping + bone cement filling + intramedullary nail internal fixation

D. Tumor segment resection + intramedullary nail internal fixation + allogeneic cortical bone ring bone grafting

E. Lesion scraping + bone cement filling

F. Chemotherapy + radiotherapy, no need for surgical treatment

201. Multiple choice questions

The patient is a 48-year-old female. She was admitted to the hospital with pain in the front of the left thigh for 1 month. The pain is dull, not relieved by rest, and not significantly aggravated by activity. She had a history of thyroid tumor resection 2 years ago, and the pathological results of the resected specimens showed that it was papillary thyroid cancer, which was well differentiated.

The recommended examination items for a clear diagnosis include (prompt physical examination: a 6cm×4cm×7cm mass can be felt in the proximal part of the left thigh, which is hard in texture, cannot be pushed, and has no tenderness.) ()

A.X-ray

B.CT

C.MRI

D.ECT

E.DSA

202. Multiple choice questions

The patient is a 48-year-old female. She was admitted to the hospital with pain in the front of the left thigh for 1 month. The pain is dull, not relieved by rest, and not significantly aggravated by activity. She had a history of thyroid tumor resection 2 years ago, and the pathological results of the resected specimens showed that it was papillary thyroid cancer, which was well differentiated.

To confirm the diagnosis, the recommended examination items include (prompt physical examination: a 5cm×7cm mass can be felt in the left thigh, which is soft in texture and non-tender.) ()

A.X-ray

B.CT

C.MRI

D.ECT

E.DSA

203, Multiple Choice Questions

The patient is a 48-year-old female. She was admitted to the hospital for 1 month due to pain in the front of the left thigh. The pain is dull, not relieved by rest, and not significantly aggravated after activity. She had a history of thyroid tumor resection 2 years ago, and the pathological results of the resected specimen showed papillary thyroid cancer, which was well differentiated.

When considering bone metastasis of thyroid cancer, diseases with similar imaging morphology of bone invasion should be excluded, including (prompt chest X-ray examination showed no abnormality. CT examination of the middle and upper segments of the left femur showed osteolytic destruction in the bone, no obvious sclerosis at the edge, unclear boundary with normal bone tissue, mild worm-like destruction and thinning of the bone cortex, and extensive involvement of the medullary cavity at the largest level. No soft tissue invasion was found. ECT showed radioactive concentration in the middle and upper segments of the left femur, and no other abnormalities were found. Blood and urine routine tests were normal, and thyroid function was normal. Thyroid ultrasound showed no tumor recurrence.) ()

A. Single myeloma

B. Intraosseous lymphoma

C. Bone tuberculosis

D. Chronic suppurative osteomyelitis

E. Fatigue fracture

204, multiple choice questions

Male, 67 years old, was diagnosed with "thyroid cancer" 3 years ago and underwent surgery. Two months ago, he felt chest and back pain without obvious cause, which did not ease after rest and worsened at night. The painkillers were ineffective. The pain gradually worsened in the past half month, making it difficult to sit and stand, and he has been bedridden. Physical examination revealed obvious tenderness in the chest and back, and normal sensation and movement in both lower limbs.

If the patient has no other complications and the lesion is relatively localized, reasonable treatment methods include ()

A. Wearing a thoracolumbar brace to reduce the pressure on the vertebral body

B. Surgical resection of the lesion

C. Taking thyroxine

D. Taking narcotic analgesics

E. Local physical therapy, pay attention to rest

205, multiple choice questions

Common bone metastasis images on X-rays include ()

A. Osteolytic bone destruction

B. Mixed bone destruction

C. Onion skin-like periosteal reaction

D. Osteogenic bone destruction

E. Parabone soft tissue mass shadow

206, multiple choice questions

The advantages of closed (puncture) biopsy are ()

A. Small surgical hematoma, less chance of tumor cell scattering

B. Small bone defect, less likely to cause pathological fractures

C. Less chance of infection

D. Suitable for areas that are difficult to reach for open biopsy, such as the spine, pelvis, etc.

E. Inexperienced physicians can also operate

207, multiple choice questions

The treatment method that is not suitable for osteochondroma is ()

A. Radiotherapy

B. Resection

C. Neoadjuvant chemotherapy

D. Amputation

E. Joint replacement

208, Multiple choice questions

Common tumors occurring in the epiphysis are ()

A. Giant cell tumor of bone

B. Osteosarcoma

C. Chondroblastoma

D. Clear cell chondrosarcoma

E. Aneurysmal bone cyst

209, Multiple choice questions

Common disease types of sacral tumors are ()

A. Chordoma

B. Giant cell tumor of bone

C. Osteosarcoma

D. Neurofibroma

E. Ewing sarcoma

210, Multiple choice questions

Treatment of non-ossifying fibroma ()

A. Surgical resection should be performed once discovered

B. Most of them tend to heal on their own and can be closely observed

C. Surgical treatment should be performed for those with persistent pain

D. In addition to surgery, radiotherapy and chemotherapy should also be used

E. Extensive resection should be performed, otherwise local recurrence is likely

211, Multiple choice questions

Which of the following statements about bone fibrosarcoma is wrong? ()

A. Bone fibrosarcoma is mostly a secondary lesion

B. Tumor bone-like tissue can be seen in pathological examination

C. It mostly occurs in adolescents

D. Imaging examination is not specific

E. Lung metastasis is prone to occur

212, multiple choice questions

Which of the following statements about the treatment of musculoskeletal system sarcoma is wrong? ()

A. Local radical cure can be achieved by amputating the diseased limb

B. Combined treatment with surgery as the main method should be adopted

C. Patients with good chemotherapy effects can be exempted from surgery

D. The only purpose of chemotherapy is to improve the limb salvage rate

E. Sarcoma will not metastasize to the lungs after radical resection

213, multiple choice questions

Which of the following statements is correct about artificial joint prostheses used for limb-saving treatment? ()

A. Tumor prostheses are generally divided into customized prostheses and modular prostheses according to the manufacturing method

B. Artificial prosthesis reconstruction is generally not used in children

C. Artificial prostheses can be reconstructed with allogeneic bone transplantation

D. Artificial prostheses have better long-term functions

E. Artificial prostheses have complications such as infection

214, Multiple Choice Questions

Which of the following statements is correct about malignant fibrous histiocytoma? ()

A. It is rare in patients under 20 years old

B. X-ray findings are specific

C. In histopathology, spindle cells are arranged in a special pinwheel or storiform pattern

D. Malignant fibrous histiocytoma is mostly a secondary lesion

E. Immunohistochemistry has diagnostic significance

215, Multiple Choice Questions

For osteosarcoma, the following statements are correct ()

A. It is common in adolescents

B. It often occurs in the metaphysis of long bones of the limbs

C. Those over 40 years old have a better prognosis

D. Pathological fractures are often the reason for medical treatment

E. Open biopsy is often recommended to confirm the diagnosis

216, multiple choice questions

The indications for limb-salvage treatment of malignant bone tumors include ()

A. Good soft tissue conditions

B. The main neurovascular bundles are not invaded, and the tumor can obtain the best resection margin

C. Local infection

D. The patient actively requests limb-salvage treatment

E. The tumor recurs multiple times

217, multiple choice questions

Common drugs for chemotherapy of osteosarcoma are ()

A. Doxorubicin

B. Cisplatin

C. Methotrexate

D. Ifosfamide

E. Fluorouracil

218, multiple choice questions

Bone metastasis cancer often originates from ()

A. Lung

B. Kidney

C. Breast

D. Liver

E. Thyroid

219, multiple choice questions

The significance of surgical treatment of bone metastasis is ()

A. Relieve pain

B. Restore walking ability

C. Facilitate care

D. Improve quality of life

E. Indirectly improve survival rate

220, multiple choice questions

The process of bone metastasis formation includes ()

A. Tumor cells detach from the primary focus

B. Tumor cells enter the surrounding lymphatic vessels or blood vessels

C. Tumor cells spread to distant sites

D. Tumor cells attach to the vascular endothelium and enter the tissue

E. Tumor cells form new lesions in the tissue

221. Multiple choice questions

Which of the following is correct about the adverse reactions of opioids ()

A. Nausea and vomiting often occur in the early stage of medication

B. Constipation is temporary

C. Drowsiness is temporary

D. Can cause respiratory depression

E. Will not cause respiratory depression

222. Multiple choice questions

Which of the following options is correct about the clinical characteristics of osteosarcoma ()

A. Osteosarcoma is prone to occur in the distal femur, proximal tibia and proximal humerus where the epiphyses of the limbs grow fastest

B. Once pathological diagnosis of osteosarcoma is confirmed, amputation should be performed immediately

C. Most osteosarcomas are single

D. Osteosarcoma also occurs more frequently in the spine and pelvis

E. Osteosarcoma can skip metastasis in bones

223. Multiple choice questions

Which of the following statements about vertebral hemangioma is correct? ()

A. Hemangioma can affect multiple vertebrae

B. Vertebral hemangioma can cause compression of the spinal cord and nerve roots and cause neurological symptoms

C. Characteristic X-ray manifestations are fence-like or grid-like images

D. Vertebral hemangioma will not affect the lamina

E. MRI shows high signals in both T and T weighted images

224, Multiple Choice Questions

The characteristics of synovial chondromatosis include ()

A. More common in large joints

B. More common in small joints

C. Pain during joint movement

D. Joint locking

E. X-ray shows multiple loose bodies in the joint, accompanied by calcification

225, Multiple Choice Questions

The patient is a 12-year-old male. The main reason was a collision while playing basketball at school, sudden left humeral pain, and fear of movement and hospitalization. No previous medical history. The fracture healed after 1 month. How to further treat the lesion ()

A. No treatment is needed for the time being, continue observation, and have regular follow-up

B. Inject steroids into the lesion and have regular follow-up

C. Scrape and bone grafting of the lesion

D. Amputation

E. After high-dose chemotherapy, resection of the tumor segment and prosthesis replacement

226. Multiple choice questions

The patient is a 22-year-old male. The main cause is pain and swelling in the lower end of the right thigh for 1 month. The examination that can be performed before surgery to evaluate the effect of chemotherapy (prompt biopsy confirmed osteosarcoma in the lower part of the right femur, and 2 courses of chemotherapy were given to prepare for surgical treatment.) ()

A. Blood routine

B. ALP

C. B-type ultrasound

D. X-ray film

E. CT, MRI

F. PET-CT

227. Multiple choice questions

The patient is a 22-year-old male. The main cause is pain and swelling in the lower end of the right thigh for 1 month. The surgical method to be used is (indicating that the preoperative chemotherapy evaluation has a good response, the tumor has shrunk, and a bone shell has formed around it.) ()

A. Thigh amputation

B. Hip disarticulation

C. Hemipelvic amputation

D. Tumor scraping and bone grafting internal fixation

E. Tumor margin resection and artificial prosthesis replacement

F. Tumor extensive resection and artificial prosthesis replacement or allogeneic bone transplantation

228, multiple choice questions

The patient is a 45-year-old male. He was admitted to the hospital for half a year because of swelling and pain in the left calf after walking. The left calf was obviously swollen after standing for a long time and walking, and there was pain from time to time, which was relieved after rest. The possible diagnosis of this case includes (prompt physical examination: T36.8℃, P82 times/min, R19 times/min, Bp150/100mmHg. The left calf mid-section is slightly bulging, and a cystic mass can be palpated in the soft tissue on the lateral side of the left tibia, about 6cm×3cm, with unclear boundaries, obvious pulsation, no obvious tenderness, local skin temperature increased, no varicose superficial veins, and no obvious decrease in sensory muscle strength in both lower limbs.) ()

A. Hemangioma

B. Giant cell tumor of bone

C. Arteriovenous malformation

D. Hematoma

E. Aneurysmal bone cyst

229, Multiple choice questions

The patient is a 45-year-old male. He was admitted to the hospital for half a year because of swelling and pain in the left calf after walking. The left calf was obviously swollen after standing for a long time and walking, and there was pain from time to time, which was relieved after rest. Further examinations and treatments that may be needed include (prompt ultrasound examination showed a soft tissue mass in the left anterior calf muscle with unclear boundaries, about 8cm×4cm×3cm in size, and abundant blood flow in the mass. CT examination showed a low-density mass on the lateral side of the left tibia with unclear boundaries and involving the tibial cortex. MRI examination T-weighted images showed mixed low signals, T-weighted images showed moderately increased signals, and the tibial cortex was eroded and destroyed.) ()

A. Angiography

B. Mass vascular embolism

C. Puncture biopsy

D. Chemotherapy

E. Radiotherapy

230, multiple choice questions

The patient is a 45-year-old male. He was admitted to the hospital for half a year due to swelling and pain in the left calf after walking. The left calf was obviously swollen after standing for a long time and walking, and there was pain from time to time, which was relieved after rest. The three examination items that should be prioritized for a clear diagnosis are ()

A. Chest X-ray

B. Left calf anteroposterior and lateral X-ray

C. Left calf ultrasound examination

D. Liver function, kidney function

E. CT

F. ECT

231. Multiple choice questions

The patient is a 66-year-old female with right hip pain for 2 months, which worsens during activity and at night. Other treatments needed include (indicating that the patient recovered well after surgery, and bone scans showed multiple rib concentrations.) ()

A. Local radiotherapy

B. Systemic chemotherapy

C. Biphosphonate drug therapy

D. Radionuclide therapy

E. Analgesic therapy

232, Multiple choice questions

Which of the following statements is wrong ()

A. Neurofibroma and schwannoma are easy to distinguish in diagnosis

B. Neurofibromatosis often has typical clinical manifestations

C. Schwannoma often presents with skin café au lait spots

D. Long-spacing collagen is a histological diagnostic indicator in both benign and malignant schwannoma

E. Benign and malignant schwannoma can be initially distinguished by clinical symptoms

233, Multiple choice questions

Male, 11 years old. Pain after left lower limb trauma for 10 days, aggravated with limited activity and fever for 3 days, admitted to hospital. It was due to a left thigh injury while running 10 days ago, obvious pain, limited activity, and slightly relieved after bed rest. The pain worsened 3 days ago, accompanied by fever, and the body temperature was as high as 40℃. He was treated with intravenous penicillin in a local hospital. X-ray examination revealed no abnormality. Physical examination: T38.8℃, P110 times/min. The circumference of the left thigh is 35cm, the circumference of the right thigh is 32cm, and the circumference of both calves is 25cm. The dorsalis pedis artery pulse is good and the skin sensation is normal. What treatment measures should be taken immediately after admission ()

A. Bed rest, immobilization of the affected limb

B. Routine examinations such as blood routine, erythrocyte sedimentation rate, liver and kidney function, and blood biochemistry

C. Systemic antibiotics

D. Systemic supportive treatment

E. Symptomatic treatment

234, multiple choice questions

Male, 15 years old. There is proximal tibia pain and swelling for 1 month, obvious pain at night, and no obvious history of trauma. The most likely diagnosis for the patient is ()

A. Bone cyst

B. Osteofibrous dysplasia

C. Aneurysmal bone cyst

D. Endochondroma

E. Osteosarcoma

F. Suppurative osteomyelitis

235, multiple choice questions

Male, 25 years old. The patient had back pain, fatigue, low fever, night sweats, and weight loss for more than 1 month, and was hospitalized after 3 days of worsening. He had been treated in other hospitals and diagnosed with "lumbar muscle strain". He was given oral analgesics and physical therapy, and his symptoms were slightly relieved. He had a poor appetite since the onset of the disease and lost 3.5kg. Physical examination: limited lumbar spine mobility, tenderness of lumbar 4-5 spinous processes, and normal sensation and movement of both lower limbs. The right lower abdomen was full, the muscles were slightly tense, and there was mild tenderness. At this time, the next treatment measure should be taken (Hint: After 3 weeks of treatment, the patient's appetite improved, lumbar pain was relieved, and there was still low fever and night sweats. ESR60mm/h, Hb100g/L were rechecked.) ()

A. Continue conservative treatment

B. Consider surgical treatment after the erythrocyte sedimentation rate returns to normal

C. Preoperative preparation should be started

D. The surgical method is anterior surgery, lesion removal, bone graft fusion

E. The surgical method is posterior surgery, lesion removal

236, multiple choice questions

Male, 25 years old. Back pain, fatigue, low fever, night sweats, and weight loss for more than 1 month, worsened for 3 days and was admitted to the hospital. He had been treated in other hospitals and diagnosed with "lumbar muscle strain". He was given oral analgesics and physical therapy, and his symptoms were slightly relieved. He has had a poor appetite since the onset of the disease and lost 3.5kg. Physical examination: limited lumbar spine mobility, tenderness of lumbar 4-5 spinous processes, and normal sensation and movement of both lower limbs. The right lower abdomen is full, the muscles are slightly tense, and there is mild tenderness. The examination that needs to be performed now is ()

A. Abdominal B-ultrasound

B. Lumbar spine anteroposterior and lateral X-ray

C. Lumbar spine CT or MRI examination

D. Liver function, kidney function, biochemistry

E. ESR, blood routine

237, multiple choice questions

Male, 53 years old, wheelchair pushed into the outpatient clinic, complaining of numbness of the right thumb and index finger, lower limb weakness, and unstable walking for 1 year without obvious inducement, which worsened for 3 weeks. Physical examination revealed increased muscle tone in the lower limbs and positive pyramidal tract signs. Which of the following is not included in the pyramidal tract signs ()

A. Hoffman sign

B. Increased muscle tone in the lower limbs

C. Oppenheim sign

D. Gorden sign

E. Kernig sign

238, Multiple choice questions

Common bone tumors in adolescent patients include ()

A. Osteochondroma

B. Osteosarcoma

C. Chondrosarcoma

D. Metastatic cancer

E. Chordoma

239, Multiple choice questions

The differential diagnosis of pigmented villonodular synovitis includes ()

A. Rheumatoid arthritis

B. Osteoarthritis

C. Joint tuberculosis

D. Trauma

E. Septic arthritis

240, Multiple choice questions

The following analgesics are not suitable for chronic cancer pain ()

A. Morphine

B. Heroin

C. Pethidine

D. Etorphine

E. Dextropropoxyphene

241, Multiple choice questions

The following descriptions of aneurysmal bone cysts are correct ()

A. It can be primary or secondary to other diseases

B. It is more common in 10 to 20 years old

C. The epiphysis of long tubular bones and the spine are the most common sites

D. The main clinical manifestations are local pain and swelling

E. X-ray shows pure osteolytic destruction and multilocular changes

242, multiple choice questions

The following are synovial tumors ()

A. Synovial sarcoma

B. Synovial cyst

C. Synovial chondromatosis

D. Pigmented villonodular synovitis

E. Soft tissue giant cell tumor

243, multiple choice questions

The following statements about NSAIDs are correct ()

A. No drug resistance

B. Dependence

C. Dose-limiting

D. Should be taken after meals

E. No dose limit

244, multiple choice questions

The types of fibrosarcoma include ()

A. Infantile fibrosarcoma

B. Adult fibrosarcoma

C. Myxofibrosarcoma

D. Low-grade malignant fibromyxoid sarcoma

E. Sclerosing epithelioid fibrosarcoma

245, Multiple Choice Questions

Diseases that need to be differentiated from ossifying fibroma ()

A. Giant cell tumor of bone

B. Dysplastic fibrous structure

C. Fibrosarcoma of bone

D. Ameloblastoma

E. Non-ossifying fibroma

246, Multiple Choice Questions

The characteristics of hereditary multiple osteochondromas are ()

A. Hereditary

B. Often associated with bone shortening or deformity

C. The rate of malignant transformation is higher than that of single-shot cases

D. There is no essential difference in histology from single-shot cases

E. As the bones mature, the tumor may stop growing

247, Multiple Choice Questions

Avoid () during biopsy

A. Transverse incision

B. Exposure of major neurovascular bundles

C. Tumor contamination of adjacent tissues

D. Careful hemostasis

E. Crushing or distorting the specimen structure

248, Multiple choice questions

The available treatments for osteolytic bone metastases of the limbs include ()

A. Chemotherapy for the primary tumor

B. Conformal radiotherapy

C. Tumor segment resection combined with internal fixation

D. Bisphosphonate therapy

E. Calcium supplementation

249, Multiple choice questions

Subtypes of liposarcoma include ()

A. Well-differentiated liposarcoma

B. Myxoid liposarcoma

C. Pleomorphic liposarcoma

D. Mixed liposarcoma

E. Dedifferentiated liposarcoma
